# Protein Kinase C Inhibitors as Modulators of Vascular Function and Their Application in Vascular Disease

**DOI:** 10.3390/ph6030407

**Published:** 2013-03-21

**Authors:** Raouf A. Khalil

**Affiliations:** Vascular Surgery Research Laboratory, Division of Vascular Surgery, Brigham and Women’s Hospital and Harvard Medical School, 75 Francis Street, Boston 02115, MA, USA; E-Mail: raouf_khalil@hms.harvard.edu; Tel.: +1-617-525-8530 (Lab: 8531); Fax: +1-617-264-5124

**Keywords:** calcium, endothelium, vascular smooth muscle, hypertension

## Abstract

Blood pressure (BP) is regulated by multiple neuronal, hormonal, renal and vascular control mechanisms. Changes in signaling mechanisms in the endothelium, vascular smooth muscle (VSM) and extracellular matrix cause alterations in vascular tone and blood vessel remodeling and may lead to persistent increases in vascular resistance and hypertension (HTN). In VSM, activation of surface receptors by vasoconstrictor stimuli causes an increase in intracellular free Ca^2+^ concentration ([Ca^2+^]_i_), which forms a complex with calmodulin, activates myosin light chain (MLC) kinase and leads to MLC phosphorylation, actin-myosin interaction and VSM contraction. Vasoconstrictor agonists could also increase the production of diacylglycerol which activates protein kinase C (PKC). PKC is a family of Ca^2+^-dependent and Ca^2+^-independent isozymes that have different distributions in various blood vessels, and undergo translocation from the cytosol to the plasma membrane, cytoskeleton or the nucleus during cell activation. In VSM, PKC translocation to the cell surface may trigger a cascade of biochemical events leading to activation of mitogen-activated protein kinase (MAPK) and MAPK kinase (MEK), a pathway that ultimately increases the myofilament force sensitivity to [Ca^2+^]_i_, and enhances actin-myosin interaction and VSM contraction. PKC translocation to the nucleus may induce transactivation of various genes and promote VSM growth and proliferation. PKC could also affect endothelium-derived relaxing and contracting factors as well as matrix metalloproteinases (MMPs) in the extracellular matrix further affecting vascular reactivity and remodeling. In addition to vasoactive factors, reactive oxygen species, inflammatory cytokines and other metabolic factors could affect PKC activity. Increased PKC expression and activity have been observed in vascular disease and in certain forms of experimental and human HTN. Targeting of vascular PKC using PKC inhibitors may function in concert with antioxidants, MMP inhibitors and cytokine antagonists to reduce VSM hyperactivity in certain forms of HTN that do not respond to Ca^2+^ channel blockers.

## 1. Introduction

Hypertension (HTN) is a major cardiovascular and renal disease affecting a large proportion of the population in the Western World. Several factors contribute to increased blood pressure (BP) including neuronal, hormonal, renal and vascular mechanisms. Understanding the physiological mechanisms that control BP would help define the pathological changes in HTN, and help design specific approaches to manage the increases in BP and HTN.

Blood vessels play a major role in the control of vascular tone. The blood vessel wall has three layers; the tunica intima made of a single layer of endothelial cells (ECs), the tunica media made of several layers of vascular smooth muscle cells (VSMCs), and the adventitia made of fibroblasts, connective tissue and extracellular matrix (ECM). ECs release vasodilator and vasoconstrictor mediators that control the vessel diameter. The ability of VSMCs to contract and relax plays an important role in the regulation of the vessel diameter and blood flow to various tissues and organs. The ECM proteins provide structural integrity to the vessel wall and are regulated by proteolytic enzymes such as matrix metalloproteinases (MMPs).

This review will focus on how vasoconstrictor agonists affect the mechanisms of VSM contraction particularly protein kinase C (PKC), and the changes in these mechanisms in vascular disease such as HTN. The role of PKC in the regulation of EC function and ECM will be briefly discussed. In addition to changes in vasoactive factors, changes in reactive oxygen species (ROS) [1,2,3], MMPs and inflammatory cytokines [4,5,6] in the plasma and vascular tissues [7,8,9] have been observed in HTN and coronary artery disease. The effects of ROS [2,3], MMPs [10,11,12] and cytokines [13,14,15] in HTN may be partly related to their effects on PKC and consequent changes in vascular reactivity, growth and remodeling. Understanding the role of PKC as a major regulator of VSM function, the PKC isoforms, their protein substrates and subcellular distribution, and their interaction with other factors such as ROS, MMPs and cytokines would provide important information regarding the benefits of determining PKC activity in the diagnosis of VSM hyperactivity disorders and the potential usefulness of PKC inhibitors in the management of vascular disease such as HTN.

## 2. Mechanisms of VSM Contraction

**Figure 1:**
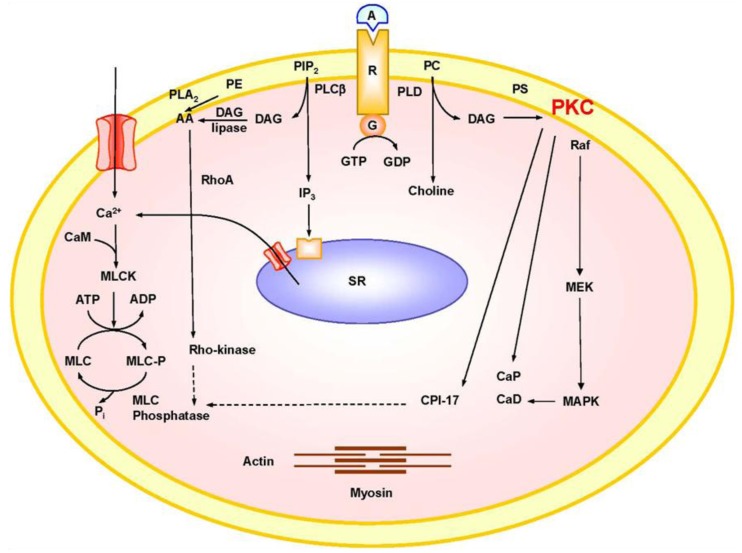
Mechanisms of VSM contraction. The interaction of an agonist (A) such as phenylephrine with its specific α-adrenergic receptor (R) activates phospholipase C (PLCβ) and stimulates the hydrolysis of phosphatidylinositol 4,5-bisphosphate (PIP_2_) into inositol-1,4,5-trisphosphate (IP_3_) and diacylglycerol (DAG). IP_3_ stimulates Ca^2+^ release from the sarcoplasmic reticulum (SR). Agonists also stimulate Ca^2+^ influx through Ca^2+^ channels. Ca^2+^ binds calmodulin (CAM), activates MLC kinase (MLCK), causes MLC phosphorylation, and initiates VSM contraction. DAG activates PKC. PKC-induced phosphorylation of CPI-17, inhibits MLC phosphatase and increases MLC phosphorylation and VSM contraction. PKC-induced phosphorylation of the actin-binding protein calponin (CaP) allows more actin to bind myosin and enhances contraction. PKC may also activate a protein kinase cascade involving Raf, MAPK kinase (MEK) and MAPK, leading to phosphorylation of the actin-binding protein caldesmon (CaD) and enhanced contraction. Activation of RhoA/Rho-kinase inhibits MLC phosphatase and further enhances the Ca^2+^ sensitivity of contractile proteins. AA, arachidonic acid; G, heterotrimeric GTP-binding protein; PC, phosphatidylcholine; PE, phosphatidylethanolamine; PLD, phopholipase D; PS, phosphatidylserine. Dashed line indicates inhibition.

Ca^2+^ is a major determinant of VSM contraction. VSM activation by physiological or pharmacological agonist triggers an increase in intracellular free Ca^2+^ concentration ([Ca^2+^]_i_) due to Ca^2+^ release from the sarcoplasmic reticulum and Ca^2+^ influx from the extracellular space through plasma membrane Ca^2+^ channels. Four Ca^2+^ ions bind to calmodulin (CAM) to form a Ca^2+^-CAM complex, which activates myosin light chain (MLC) kinase, and in turn causes the phosphorylation of the 20 kDa MLC, stimulates actin-myosin interaction and promotes VSM contraction (Figure 1). During VSM relaxation, removal of the vasoconstrictive agonist causes a decrease in [Ca^2+^]_i_ due to Ca^2+^ extrusion via the plasmalemmal Ca^2+^ pump (PMCA) and the Na^+^-Ca^2+^ exchanger, as well as Ca^2+^ reuptake via the sarcoplasmic reticulum Ca^2+^ pump (SERCA). The decrease in [Ca^2+^]_i_ allows the dissociation of the Ca^2+^-CAM complex, and the remaining phosphorylated MLC is dephosphorylated by MLC phosphatase, leading to detachment of actin-myosin crossbridges and VSM relaxation [16,17,18,19].

Ca^2+^-dependent VSM contraction is usually observed during VSM depolarization by mechanical stretch, nerve stimuli, electrical stimulation, or in the presence of high KCl solution. VSM depolarization activates voltage-gated Ca^2+^ channels (VGCCs) and because of the large concentration gradient between extracellular Ca^2+^ (millimolar) and intracellular [Ca^2+^]_i_ (nanomolar), the opening of VGCCs facilitates Ca^2+^ influx, and leads to MLC phosphorylation and VSM contraction. In contrast with membrane depolarization, physiological agonists such as norepinephrine, prostaglandin F2α and thromboxane A2 activate other intracellular signaling pathways in addition to Ca^2+^ channels. In VSM, the interaction of an agonist with its specific receptor causes activation of phospholipase C (PLC), and promotes the hydrolysis of phosphatidylinositol 4,5-bisphosphate into inositol 1,4,5-trisphosphate (IP_3_) and diacylglycerol (DAG) [20,21]. Because IP_3_ is water soluble it diffuses in the cytosol, stimulates IP_3_ receptors in the sarcoplasmic reticulum and causes Ca^2+^ release from the intracellular stores, transient increase in [Ca^2+^]_i_ and VSM contraction. Agonists also activate receptor-operated (ROCs) and store-operated Ca^2+^ channels (SOCs), causing maintained Ca^2+^ influx, increased [Ca^2+^]_i_, MLC phosphorylation and VSM contraction (Figure 1). However, Ca^2+^-dependent MLC phosphorylation may not be the only mechanism involved in VSM contraction. For instance, Ca^2+^ channel blockers such as nifedipine, verapamil or diltiazem do not completely inhibit agonist-induced VSM contraction. Also, agonist-induced maintained contraction has been observed in certain blood vessels incubated in Ca^2+^-free solution and in the absence of detectable increases in [Ca^2+^]_i_ [17,22,23,24]. Agonist-induced dissociations between [Ca^2+^]_i_ and force, between [Ca^2+^]_i_ and MLC phosphorylation, and between MLC phosphorylation and force have also been observed in several vascular preparations, suggesting activation of additional signaling pathways that cause sensitization of the contractile myofilaments to [Ca^2+^]_i_ including Rho-kinase and protein kinase C (PKC) [16,18].

## 3. PKC Isoforms

**Figure 2:**
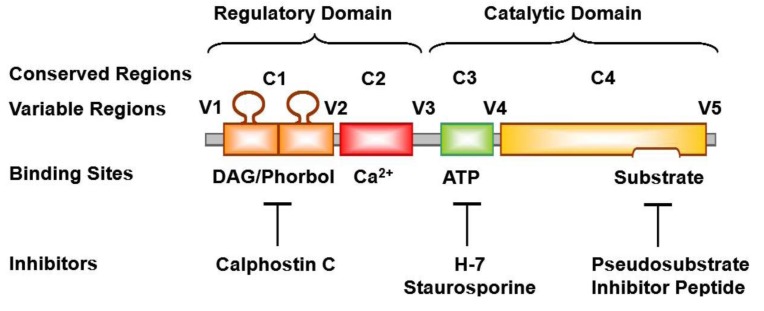
Biochemical structure of a classic PKC, and sites of action of activators and inhibitors. A classic PKC molecule has four conserved (C1–C4) and five variable (V1–V5) regions. C1 region contains binding sites for DAG, phorbol ester, and phosphatidylserine, and for the PKC inhibitor calphostin C. C2 region contains Ca^2+^-binding site. C3 regions contain binding sites for ATP and the PKC inhibitors H-7 and staurosporine. C4 region contains binding sites for physiological PKC substrate. Endogenous pseudosubstrate and exogenous pseudosubstrate inhibitor peptide bind to the catalytic domain and prevents PKC from phosphorylating the true substrate. Upon activation, the PKC molecule unfolds to remove endogenous pseudosubstrate, and bring ATP into proximity with the substrate.

PKC was first described as a Ca^2+^-activated phospholipid-dependent protein kinase [25], but subsequent biochemical analysis and molecular cloning revealed a family of different PKC isozymes of closely related structure. The PKC molecule is a single polypeptide, comprised of N-terminal regulatory domain and C-terminal catalytic domain (Figure 2) separated by a hinge region that becomes proteolytically labile when the enzyme is membrane-bound [26]. Classic PKCs have four conserved regions (C1-C4) and five variable regions (V1-V5). The C1 region contains a tandem repeat of the characteristic cysteine-rich zinc-finger-like sequence. The sequence Cys-X2-Cys-X13(14)-Cys-X7-Cys-X7-Cys, where X represents any amino acid, is conserved among different PKCs, and each 30-residue sequence of this type is independently folded and binds a zinc ion [27]. The Cys-rich motif is duplicated in most PKCs and may form the DAG/phorbol ester-binding site. The Cys-rich motif is also immediately preceded by an autoinhibitory pseudosubstrate sequence. The C1 region also contains the phosphatidylserine recognition site [26]. In Ca^2+^-dependent PKCs, the C2 region is rich in acidic residues and has a Ca^2+^-binding site. The C3 and C4 regions contain the adenosine triphosphate (ATP) and substrate binding sites. Similar to other protein kinases, all PKCs have an ATP-binding sequence, Gly-X-Gly-X-X-Gly-----Lys [21,26] (Figure 2).

PKC isoforms are generally classified into three subgroups, conventional or classic cPKs, novel nPKCs, and atypical aPKCs (Table 1). cPKCs include α, βI, βII, and γ PKC. They have the traditional 4 conserved regions (C1–C4) and the 5 variable regions (V1–V5). The cDNA clones for α, βI, βII and γ PKC were isolated from bovine [28,29], rat [30], rabbit [31] and human brain libraries [28]. βI and βII cDNAs are derived from a single mRNA transcript by alternative splicing, and differ only in ~50 amino acid residues in their carboxyl-terminal end in the variable region V5 [30,31]. Because α, βI, βII and γ-PKC have a phorbol ester biding site, they are down-regulated by prolonged exposure to phorbol esters. nPKCs include δ, ε, η(L) and θ PKC. They lack the C2 region and are Ca^2+^-independent [30]. ε-PKC differs from α-, βI-, βII-, and γ-PKC in that the V1 region is extended while the C2 region is deleted [32]. η-PKC shows the highest sequence similarity to ε-PKC with 59.4% identity [33]. PKC L, is the human homologue of mouse η-PKC [34]. θ-PKC consists of 707 amino acids and shows the highest sequence similarity to δ-PKC (67% identity) [33]. aPKCs include ζ and λ/ι PKC, and characteristically have only one Cys-rich zinc finger-like motif. aPKCs are dependent on phosphatidylserine, but are not affected by DAG, phorbol esters or Ca^2+^, and therefore do not translocate or downregulate in response to phorbol esters or DAG [30] (Table 1).

**Table 1:** Representative PKC Distribution and Subcellular Distribution in Blood Vessels.

PKC	MW (kDa)	Blood Vessel	Resting Cell	Activated Cell	Ref
**Classic**					
α	74–82	Rat aorta	Cytosolic	Nuclear	[35]
		Rat Carotid artery	Cytosolic	Membrane	[36]
		Rat mesenteric artery	Cytosolic/membrane	Cytosolic/Membrane	[37]
		Porcine coronary artery	Cytosolic	Membrane	[38]
		Bovine aorta	Cytosolic	Membrane	[39]
		Ferret portal vein	Cytosolic	Surface membrane	[40]
β	80–82	Rat aorta	Cytosolic	Nuclear	[35]
		Rat Carotid artery	Cytosolic	Membrane	[36]
γ	70–82	Rat mesenteric artery	Cytosolic	Cytosolic	[37]
**Novel**					
δ	76–82	Rat aorta	Cytoskeleton/organelle	Cytoskeleton/organelle	[41]
		Rat mesenteric artery	Membrane	Membrane	[37]
ε	90–97	Rat mesenteric artery	Cytosolic/membrane	Cytosolic/membrane	[37]
		Ferret aorta	Cytosol	Surface membrane	[42]
		Porcine coronary artery	Cytosolic	Membrane	[38]
η		NIH 3T3 fibroblasts	Cytosolic/membrane	Membrane	[43]
**Atypical**					
ζ	64–82	Rat aorta	Perinuclear	Intranuclear	[41]
		Rat mesenteric artery	Cytosolic	Cytosolic	[37]
		Ferret aorta, portal vein	Perinuclear	Intranuclear	[42]
λ/ι	70	Rabbit femoral artery	Cytosolic	Cytosolic	[44]
		Rabbit portal vein			

## 4. PKC Substrates

The inactive PKC molecule is folded such that the basic autoinhibitory pseudosubstrate is tightly bound to the acidic patch in the substrate-binding site, a conformation that protects it from proteolysis. When PKC is activated by phosphatidylserine, DAG and Ca^2+^ the pseudosubstrate becomes unmasked and liable to proteolysis. This is supported by the observation pseudosubstrate antibodies activate PKC likely by removing the pseudosubstrate from the active substrate binding site [45]. Activated PKC phosphorylates arginine-rich protein substrates, and these peptides neutralize the acidic patch that maintains the pseudosubstrate in the active site, thus displacing the basic pseudosubstrate from the substrate-binding site in the catalytic domain [26,46,47]. The amino acid sequence in the vicinity of the substrate phosphorylation site may provide a substrate recognition guide for PKC and structure-function studies of synthetic peptide substrates suggest that PKC requires basic residue determinants in common with other serine/threonine protein kinases [47].

Common PKC substrates include lysine-rich histone and myelin basic protein [25]. α-, β-, γ-, and ζ-PKC are potent histone IIIS kinases. δ-, ε-, and η-PKC do not adequately phosphorylate histone IIIS, but readily phosphorylate myelin basic protein [32,48,49]. However, removal of the regulatory domain of ε-PKC by limited proteolysis generates a catalytic fragment that can phosphorylate histone IIIS [32].

Myristoylated, alanine-rich C kinase substrate (MARCKS) is an 87-kDa protein and a major PKC substrate that binds F-actin and bridges cytoskeletal actin to the plasma membrane [50,51]. Also, PKC-induced phosphorylation of the inhibitory GTP-binding protein G_i_ facilitates the dissociation of its α_i_ subunit from adenylyl cyclase and leads to increased adenylyl cyclase activity [52]. Other PKC substrates include plasma membrane ion channels and pumps. PKC inhibits Ca^2+^-dependent large conductance K^+^ channel (BK_Ca_) in pulmonary VSM [53]. Also, thromboxane A_2_ may inhibit voltage-gated K^+^ channels and pulmonary vasoconstriction via a mechanism involving ζ-PKC [54]. PKC-induced phosphorylation of SERCA promotes Ca^2+^ uptake, and activation of PMCA promotes Ca^2+^ extrusion, leading to reduction in agonist-induced increase in VSM [Ca^2+^]_i_ [55]. PKC may phosphorylate the α1 subunit of Na^+^/K^+^-ATPase, and activate the Na^+^/H^+^ exchanger and thereby increase cytoplasmic pH and cause cell alkalinization [56,57].

PKC substrates also include cytoskeletal and regulatory proteins in VSM. PKC-induced phosphorylation of vinculin, a cytoskeletal protein localized at adhesion plaques, could affect cell shape and adhesion properties [58]. PKC also causes phosphorylation of the CPI-17 regulatory protein leading to inhibition of MLC phosphatase, increased MLC phosphorylation and enhanced VSM contraction [59]. Also, α-PKC phosphorylates the actin-binding protein calponin, allowing more actin to interact with myosin and further VSM contraction [29]. However, PKC could also phosphorylate the 20-kDa MLC and MLC kinase, leading to inhibition of Ca^2+^-dependent actin-myosin interaction and VSM contraction [60].

## 5. PKC Distribution

PKC isoforms are expressed in various vascular beds (Table 1). α-PKC is a universally expressed in almost all blood vessels examined. γ-PKC is expressed mainly in neurons and nerve endings of blood vessels. δ-PKC is mainly associated with the cytoskeleton. ζ-PKC is universally expressed in many vascular tissues. η/L-PKC is expressed in the lung, skin, heart and brain, θ-PKC in skeletal muscle and ι/λ-PKC in the testis and ovary [49].

In resting cells, α, β and γ-PKC are localized mainly in the cytosolic fraction, and activated PKC undergoes translocation from the cytosolic to the particulate and membrane fraction [61,62]. Activated α-, β- and γ-PKC usually undergo translocation from the cytosol to the cell membrane [63] (Table 1). However, in normal fibroblasts, α-PKC is tightly associated with the cytoskeleton and organized into plasmalemmal focal contacts which are composed of structural proteins such as vinculin, talin, integrin and α-actinin that allow the attachment of cytoskeletal microfilaments to the plasma membrane [64]. In neural cells, βI-PKC is associated with the plasma membrane, while βII-PKC is localized in the Golgi complex [21]. In the cerebellum, γ-PKC is present in the cell bodies, dendrites and axons of Purkinje’s cells. Immuno-electron microscopy revealed that γ-PKC is associated with most cell membranous structures, except the nucleus [65].

Because δ-PKC is localized in the vicinity of the cytoskeleton, it is often identified in the particulate fraction of both resting and activated cells. In contrast, ε-PKC undergoes translocation from the cytosol to the surface membrane during VSM activation. ζ-PKC is localized in the vicinity of the nucleus in both resting and activated mature VSMCs [42]. However, in the developing embryo, ζ-PKC may have different distribution and function and may play a role in perinatal pulmonary vasoconstriction [66].

Different physico-chemical forces may drive PKC translocation including simple diffusion or specific targeting mechanisms that allow tight binding of PKC to its target substrate. Some of the targeting mechanisms include conformation changes and altered hydrophobicity, lipid modification, phosphorylation and targeting sequences. For instance, binding of Ca^2+^ or DAG to PKC may cause conformational changes that unfolds the PKC molecule and result in exposure of the substrate region and increased PKC hydrophobicity and binding to membrane lipids [26]. Also, modification in the lipid component of a protein could influence its subcellular distribution. The VSM plasma membrane is composed of several domains of focal adhesions alternating with zones rich in caveolae, and both harbor a subset of membrane-associated proteins. Also, the plasma membrane lipids are segregated into cholesterol-rich lipid rafts and glycerophospholipid-rich non-raft regions, an arrangement that is critical for preserving the membrane protein architecture and for the translocation of proteins to the plasma membrane. In VSMC membrane, lipid segregation is supported by annexins that target membrane sites of distinct lipid composition, and each annexin requires different [Ca^2+^] for its translocation to the plasma membrane, thus allowing a spatially confined graded response to external stimuli and plasmalemmal localization of PKC [67]. Protein phosphorylation could also change their conformation or electric charge and consequently affect their lipid affinity and binding to the plasma membrane. While myristoylation of MARCKS is essential for its binding to actin and the plasma membrane, its phosphorylation by PKC may have an electrostatic effect that affects the protein affinity to the plasma membrane and consequently interferes with its actin cross-linking and causes its displacement from the plasma membrane. This is supported by the observation that dephosphorylation of MARCKS causes its re-association with the plasma membrane via its stably attached myristic acid membrane-targeting moiety [68]. Phosphorylation of PKC itself via autophosphorylation or by a putative PKC kinase may also determine its localization and full activation, and PKC phosphorylation sites have been identified in the catalytic domain of α-, β- and δ-PKC [69]. Also, binding sites for arginine-rich polypeptides have been identified in the PKC molecule distal to its catalytic site allowing targeting of PKC to target substrates at specific subcellular locations [70]. Receptors for activated C-kinase (RACKs) may target PKC to cytoskeletal elements, while a peptide inhibitor derived from the PKC binding proteins annexin I and RACKI may interfere with translocation of β-PKC [71].

## 6. PKC Function

PKC is involved in many physiological functions including secretion and exocytosis, modulation of ion channel, gene expression and cell growth and proliferation [21,49]. For example, transfection of a vector containing the full-length cDNA encoding βI-PKC in rat fibroblasts led to overexpression of the isozyme and caused cell growth abnormalities that mimicked the effects of the tumor promoter phorbol esters. However, these cell lines did not exhibit the typical characteristics of malignantly transformed fibroblasts. Hence, the overproduction of PKC *per se* may not be sufficient to cause cancer, although it may facilitate the cell conversion to malignancy by genotoxic agents [72].

PKC may exert negative-feedback control over cell signaling by downregulation of surface receptors and/or inhibition of agonist-induced activation of PLC and phosphoinositide hydrolysis [21]. Also, PKC may play a role in VSM contraction [18,21,49,73]. PKC activators such as DAG analogs and phorbol esters cause contraction in isolated blood vessels *ex vivo* [17,18,49]. Phorbol ester-induced vascular contraction is not associated with detectable increases in [Ca^2+^]_I_, and a role of Ca^2+^-independent ε-PKC has been suggested [24,42]. Also, PKC inhibitors inhibit agonist-induced contraction of coronary VSM [17,73]. However, PKC may induce phosphorylation of MLC kinase leading to inhibition of VSM contraction [60].

PKC-induced phosphorylation of certain substrates may activate a cascade of protein kinases that enhance VSM contraction [74]. PKC-induced phosphorylation of CPI-17 promotes the inhibition of MLC phosphatase and leads to further increases in MLC phosphorylation and VSM contraction (Figure 1) [59]. α-PKC-induced phosphorylation of the actin binding protein calponin could reverse the calponin-mediated inhibition of actin-activated myosin ATPase, thus allowing more actin to interact with myosin and enhance VSM contraction (Figure 1) [18,29].

Mitogen-activated protein kinase (MAPK) is a Ser/Thr protein kinase that requires dual phosphorylation at both the Thr and Tyr residues for its activation. In quiescent undifferentiated VSMCs, MAPK is mainly in the cytosol, but upon cell activation by a growth factor or a mitogen, MAPK undergoes translocation from the cytosol to the nucleus where it promotes gene expression and cell growth [75]. Importantly, tyrosine kinase and MAPK activities have been identified in differentiated contractile VSM, suggesting a role in VSM contraction [74]. Activation of differentiated VSMCs with the α-adrenergic agonist phenylephrine is associated with an initial translocation of MAPK from the cytosol to the surface membrane. However, during maintained VSM activation MAPK undergoes redistribution from the surface membrane to the cytoskeleton [74]. It is likely that agonist-induced activation and generation of DAG at the surface membrane promotes translocation of the Ca^2+^-independent ε-PKC from the cytosol to the surface membrane, where it becomes fully activated. Activated ε-PKC in turn promotes translocation of both MAPK kinase (MEK) and MAPK from the cytosol to the surface membrane to form a protein kinase complex. PKC then induces phosphorylation and activation of MEK, which in turn causes phosphorylation of MAPK at both Thr and Tyr residues [76]. Tyr-phosphorylated MAPK is then targeted to the cytoskeleton, where it induces phosphorylation of the actin-binding protein caldesmon [77,78]. The phosphorylation of caldesmon reverses its inhibition of actin-mediated MgATPase activity leading to further increases in actin-myosin crossbridge cycling and VSM contraction (Figure 1) [18,74].

## 7. PKC Activators

PKC isoforms have different sensitivity to Ca^2+^, phosphatidylserine, DAG and other phospholipid products. Ca^2+^-dependent PKCs bind Ca^2+^ in a phospholipid-dependent manner such that Ca^2+^ may form a “bridge” holding the PKC-phospholipid complex at the plasma membrane [79]. Phosphatidylserine is required for activation of most PKCs. Phosphatidylinositol and phosphatidic acid may activate PKC, but may require high Ca^2+^ concentrations. DAG activates Ca^2+^-independent PKCs and reduces the Ca^2+^ requirement for activation and membrane association of Ca^2+^-dependent PKCs [21].

Lipids derived from sources other than glycerolipid hydrolysis such as *cis*-unsaturated free fatty acids and lysophosphatidylcholine, ceramide (a sphingomyelinase product), phosphatidylinositol 3,4,5-trisphosphate and cholesterol sulfate may also activate PKC [80]. Other PKC activators include phorbol esters such as 12-o-tetradecanoylphorbol-13-acetate (TPA), phorbol myristate acetate (PMA) and phorbol 12,13-dibutyrate (PDBu). Phorbol esters reduce the apparent K_m_ of PKC for Ca^2+^ and stabilize it in the membrane-bound form [49].

Bryostatin, a marine natural product, binds to and activates PKC and is more potent than PMA in translocating δ- and ε-PKC but is not a carcinogen or a complete tumor promoter [81]. Oxidized low density lipoprotein (LDL) increases the activity of α- and ε-PKC in coronary VSM, and promotes coronary artery vasoconstriction and atherogenesis [82]. γ-Radiation may activate α- and ε-PKC, and in turn promote smooth muscle cell apoptosis [83].

PKC activity and affinity for its substrate could be modified by its phosphorylation by other protein kinases or even by its own autophosphorylation [84,85,86]. α-, βI- and βII-PKC are expressed as inactive precursors that require phosphorylation by a “PKC kinase” for permissive activation. Phosphorylation of α-PKC may prevent its down-regulation during prolonged exposure to phorbol ester [85]. Also, phosphorylation of βII-PKC at the C-terminus allows it to bind ATP and substrate with higher affinity. Phosphorylation of structure determinants in the regulatory domain of PKC may increase its affinity to Ca^2+^ [86]. Autophosphorylation of the Ca^2+^-independent δ-PKC at Ser-643 may occur *in vivo*, and consequently control the activity and biological function of δ-PKC [84].

## 8. PKC Inhibitors

Several PKC inhibitors with different affinity, efficacy and specificity have been developed (Table 2). PKC inhibitors acting on the catalytic domain by competing with ATP are not specific and inhibit other protein kinases. PKC inhibitors acting on the regulatory domain by competing at the DAG/phorbol ester or the phosphatidylserine binding site may be more specific. While prolonged exposure to phorbol esters can downregulate α-, β-, γ-, and ε-PKC [38], the tumor promoting actions of phorbol esters limit their use.

The pseudosubstrate region in the regulatory domain of PKC contains an amino acid sequence between the 19 and 36 residues that resembles the substrate phosphorylation site. Synthetic pseudosubstrate inhibitor peptides (19 to 36) inhibit specific PKCs by exploiting their substrate specificity without interfering with ATP binding. These synthetic peptides inhibits both PKC substrate phosphorylation and PKC autophosphorylation [47]. Also, myr-ψPKC, a myristoylated peptide based on the substrate motif of α- and β-PKC, inhibits TPA-induced PKC activation and phosphorylation of MARCKS [87].

In smooth muscle, α-tocopherol inhibits the expression, activity and phosphorylation of α-PKC, while β-tocopherol protects PKC from the inhibitory effects of α-tocopherol [88].

Short interference RNA (siRNA) can prevent the expression of a specific PKC isoform and thereby determine its role in a specific cellular function. Antisense techniques, knockout mice and transgenic animals have also been used to study the effects of downregulation of a specific PKC isoform *in vivo*.

**Table 2:** Representative PKC Inhibitors.

Chemical Group	Example	Site of Action	Specificity
1-(5-isoquinolinesulfonyl)-2-methylpiperazines	H-7	Catalytic domain Compete with ATP at the ATP binding site	Also, inhibits cyclic AMP and cyclic GMP-dependent protein kinases
Microbial Alkaloids, Products of Streptomyces	Staurosporine SCH47112	Catalytic domain, ATP binding site	Also, inhibits MLC kinase and tyrosine kinase
Benzophenanthridine Alkaloids	Chelerythrine	Catalytic domain	Competitive inhibitor with histone IIIS
Indocarbazoles	Gö6976	Catalytic domain	Ca^2+^-dependent α- and βI-PKC
Bisindolylmaleimide Staurosporine Analogs	GF109203X Ro-318220 Midostaurin (PKC412, CGP41251) Ruboxistaurin (LY333531)	Catalytic domain	PKC isozymes α, βI, βII, γ, δ and ε. Ruboxistaurin mesylate is a selective antagonist of PKC βI and PKC βII.
Perylenequinone Metabolites from Cladosporium cladosporioides	Calphostin C (UCN-1028A)	Regulatory domain	Binds to the regulatory domain at DAG/phorbol ester binding site
Membrane lipids	Sphingosine	Regulatory domain	Competitive inhibitor with phosphatidylserine
Other:	Adriamycin Aminoacridine Apigenin Cercosporin Chlorpromazine Dexniguldipine Polymixin B Sangivamycin Tamoxifen Trifluoperazine UCN-01, UCN-02		

## 9. PKC and Hypertension

**Figure 3:**
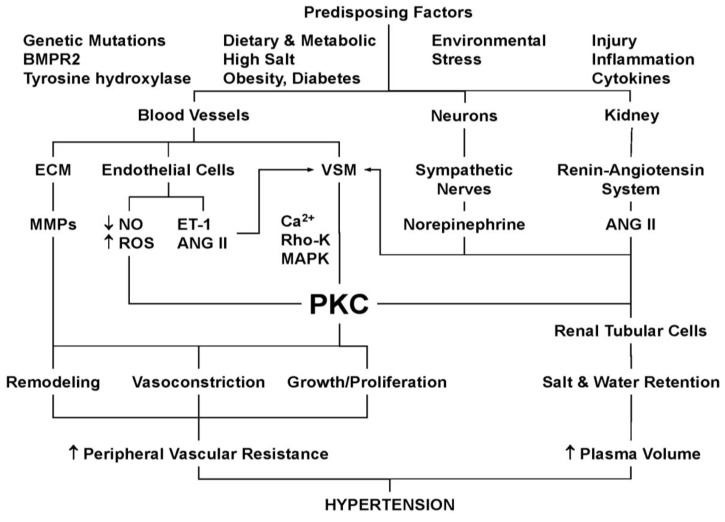
Role of PKC in hypertension. Genetic, dietary and environmental risk factors, lead to vascular, neural and renal dysfunction, and increased release of various mediators from endothelial cells (ROS, ET-1, ANG II), sympathetic neurons (norepinephrine) and the kidney (ANG II). These mediators could stimulate VSM and activate PKC, as well as Ca^2+^, Rho kinase, and MAPK thereby induce vasoconstriction and VSM growth and proliferation. The interaction of PKC with matrix metalloproteinases (MMPs) in the extracellular matrix (ECM) could contribute to vascular remodeling. Activation of the renin-angiotensin system (RAS) and increased ANG II production induce salt and water retention and increase plasma volume. Persistent increases in peripheral vascular resistance and plasma volume lead to hypertension.

HTN is a multifactorial disorder that involves changes in the neural, hormonal, renal and vascular control mechanisms of BP [89]. Increases in the amount and activity of PKC could cause disturbance in one or more of these physiological control mechanisms, leading to persistent increases in BP and HTN. PKC could promote VSM growth, proliferation and contraction pathways. The relation between PKC and HTN could also involve changes in the vascular endothelium, ECM and MMPs-mediated vascular remodeling, oxidative stress and free radicals, renal hemodynamics and renin-angioensin system, neuronal changes and sympathetic hyperactivity, vascular inflammation and potential interactions with inflammatory cytokines, and other metabolic factors (Figure 3).

## 10. PKC and VSM Growth and Reactivity in Hypertension

Increased expression/activity of PKC could promote trophic changes in VSM and lead to increases in the vessel wall thickness and hypertrophic remodeling (Figure 3). Overexpression of α-PKC in A7r5 VSMC line stimulates cell proliferation [90]. Also, the localization of ζ-PKC in the vicinity of the nucleus suggests a role in VSM growth and the hypertrophic remodeling associated with HTN [41,42]. Combined increases in PKC activity and [Ca^2+^]_i_ could exert trophic effects in both the vasculature and the heart, leading to narrowing of the arterial lumen and cardiac hypertrophy in long-standing HTN [57]. Increased PKC expression and activity could also enhance vasoconstriction and increase vascular resistance and BP (Figure 3). α-PKC activation has been shown to enhance VSM contraction, and its overexpression in VSM may be involved in HTN [41,42]. Also, the Ca^2+^-independent ε-PKC may enhance the myofilament force sensitivity to [Ca^2+^]_i_ in VSM and promote the vasoconstriction associated with HTN [18,42]. The localization of δ-PKC in the cytoskeleton suggests that it may play a role in the vascular remodeling observed in HTN [49].

## 11. PKC in Genetic Hypertension

Genetic linkage studies in certain families have supported the genetic origin of HTN. For instance, mutations in BMPR2 gene, which encodes a bone morphogenetic protein receptor II, a TGF-β superfamily member, have been linked to 55% of familial pulmonary arterial HTN [91,92,93]. Mice carrying BMPR2 heterozygous alleles (BMPR2^+/−^) are genetically equivalent to mutant human gene and develop pulmonary arterial HTN under stress conditions [94]. Proteomics studies on mouse tissues have identified β-PKC as one of the signaling pathways associated with BMPR2 [95], suggesting a role of PKC in genetic HTN.

PKC may also play a role in spontaneously hypertensive rats (SHR). Norepinephrine-induced contraction is more readily inhibited by the PKC inhibitor 1-(5-isoquinolinesulfonyl)-2-methylpiperazine (H-7) in the aorta of SHR than Wistar-Kyoto rats (WKY). Also, treatment of the aortic segments with H-7 causes a shift to the right in the concentration-contraction curve of the PKC activator TPA in the aorta of SHR, but not WKY [96]. The PKC activator PDBu also produces contraction and greater reduction in cytosolic PKC in the aorta of SHR than WKY [97]. In SHR, γ-interferon restores PKC level to that in normal control rat, suggesting an interaction between PKC and cytokines in genetic HTN [98].

The role of PKC in genetic HTN has been further studied by measuring vascular contraction and PKC activity during the development of HTN in young (5–6 weeks) SHR. High KCl-induced contraction in intact mesenteric arteries and the Ca^2+^-force relationship in vessels permeabilized with α-toxin were not different in SHR and WKY rats. Treatment with the PKC activator PDBu caused greater enhancement of high KCl-induced contraction in intact vessels and the Ca^2+^-force relationship in permeabilized vessels of SHR than those of WKY. The PKC inhibitors H-7 and calphostin C caused greater inhibition of contraction in blood vessels of SHR than WKY. These data support that PKC enhances the Ca^2+^ sensitivity of the contractile proteins in VSM to a greater extent in blood vessels of young prehypertensive SHR than WKY. The data also suggest that PKC activation in VSM occurs before overt HTN, and support a causative role of PKC in the development of genetic HTN [99].

To further examine potential inborn differences in vascular PKC before the onset of HTN, studies have compared VSM proliferation in cells from young (1–2 week) SHR and WKY rats. In cultured aortic VSM from SHR and WKY rats, both ANG II and endothelin-1 (ET-1) enhanced thymidine incorporation into DNA, an indicator of DNA synthesis. Treatment of VSMCs with the PKC inhibitor chelerythrine caused greater suppression of ANG II and ET-1 induced DNA synthesis and VSM growth in cells of SHR than WKY, suggesting an inborn increase in PKC activity in VSMCs of SHR [100].

In a study assessing the role of PKC in the changes in vascular tone associated with genetic HTN *in vivo*, it was found that perfusing the PKC activator PDBu in the hindlimb of anesthetized SHR and WKY rats caused prolonged vasoconstriction and increased perfusion pressure. The PDBu-induced vasoconstriction and increased perfusion pressure were inhibited by the PKC inhibitor staurosporine to a greater extent in SHR than WKY rats, supporting a role of PKC in the regulation of vascular function and BP *in vivo*, and increased PKC expression and activity in VSM of rat models of genetic HTN [101].

Sex differences in the expression and activity of PKC have been observed in VSM of WKY and SHR. VSM contraction and the expression and activity of α-, δ- and ζ-PKC in response to the phorbol ester PDBu are less in intact female than intact male WKY, and these sex differences are greater in VSM from SHR than WKY rats [102]. PDBu-induced contraction and PKC activity were similar in castrated and intact male rats, but greater in ovariectomized (OVX) than in intact female rats. Treatment of OVX females with 17β-estradiol subcutaneous implants caused reduction in PDBu contraction and PKC activity, that were greater in SHR than WKY rats. These data suggested sex-related reduction in VSM contraction and the expression and activity of α-, δ- and ζ-PKC in female compared with male rats, and that these differences are likely mediated by estrogen and are enhanced in genetic HTN [102].

## 12. PKC and Human Essential Hypertension

Studies have shown an increase in oxidative stress and growth response in VSMCs from resistance arteries of patients with essential HTN as compared to cells from normotensive controls. ANG II increases ROS to a greater extent in VSM from hypertensive than normotensive subjects. Also, ANG II increases phospholipase D (PLD) activity and DNA and protein synthesis to a greater extent in VSMCs from hypertensive than normotensive subjects, and the ANG II effects are partially inhibited by treating the cells with the PKC inhibitors chelerythrine and calphostin C. These data suggest that the increased oxidative stress and growth-promoting effects of ANG II in VSMCs from hypertensive patients may involve increased activity of PLD- and PKC-dependent pathways and further support a role of these pathways in the vascular remodeling associated with HTN [103].

One of the properties of PKC is that it undergoes translocation from the cytosol to cell membrane during VSM activation, a property that can be used in the diagnosis and prognosis of VSM hyperactivity in HTN. However, the subcellular distribution of PKC may vary depending on the type and abundance of membrane lipids. Studies have shown increased cholesterol/phospholipid ratio, higher levels of monounsaturated fatty acids, and lower levels of polyunsaturated fatty acids in erythrocyte membranes from elderly hypertensive subjects as compared to normotensive controls. On the other hand, the levels of activated membrane-associated PKC are not increased, but rather decreased in erythrocytes of elderly hypertensive subjects, which may not be related to the etiopathology of HTN, but represent an adaptive compensatory mechanism to HTN [104].

## 13. PKC and Aortic Constriction-Induced Hypertension

PKC activation and translocation are increased in a rat model of pressure overload and left ventricular hypertrophy produced by banding or clipping of the aorta [41]. The increased PKC activity is associated with increased tritiated phorbol ester ([^3^H]PDBu) binding and PKC concentration in both the cytosolic and membrane fractions [105]. Immunoblot analysis has revealed that the increased PKC activity is mainly due to increases in the amount of βI-, βII- and ε-PKC in the surface membrane and nuclear-cytoskeletal fractions [105]. Imaging of the subcellular distribution of PKC revealed that in VSMCs of normotensive rats α-PKC is mainly in the cytosol, while ζ-PKC is in the perinuclear area [40,42]. In VSMCs of hypertensive rats, α-PKC is activated and localized at the surface membrane, while ζ-PKC is localized in the nucleus [41].

## 14. PKC, Endothelial Dysfunction and Hypertension

Changes in PKC activity in the endothelium could contribute to the regulation of vascular function and BP. Studies have suggested a role of PKC in the endothelial cell dysfunction observed in blood vessels of SHR and deoxycorticosterone acetate (DOCA)-salt hypertensive rats [106,107]. NO is one of the major vasodilators produced by the endothelium. Activated endothelial NO synthase (eNOS) catalyzes the transformation of l-arginine to l-citrulline and the production of NO. Mice deficient in eNOS are hypertensive and lack NO-mediated vasodilation [108]. PKC activation may affect NOS activity and NO production or bioactivity. PKC may cause phosphorylation of Thr-495 and dephosphorylation of Ser-1175 in eNOS and in turn inhibit NO production [109,110]. Specifically, α- and δ-PKC phosphorylate eNOS at Ser-1175 and increase NO production [111,112]. PKC may also play a role in eNOS “uncoupling”, a process in which eNOS is over-expressed or hyperactivated in an attempt to produce more NO to reduce vascular tone, but instead produces superoxide (O_2_^−^•) [113,114]. In SHR, oral administration of the PKC inhibitor midostaurin, a staurosporine analog, reverses aortic eNOS “uncoupling”, and causes up-regulation of eNOS expression and diminished production of ROS. Also, aortic levels of (6R)-5,6,7,8-tetrahydro-L-biopterin (BH4), a NOS cofactor, are reduced in SHR compared with WKY. In addition, midostaurin lowered BP in SHR and, to a lesser extent in WKY [115], supporting potential benefits of PKC inhibitors in genetic HTN.

## 15. PKC, Oxidative Stress and Hypertension

Oxidative stress has been demonstrated in most forms of HTN including essential and renovascular HTN. Increased O_2_^−^•production decreases NO bioactivity, and in turn increases vasoconstriction and vascular resistance in HTN [1,2,3]. The HTN-associated increase in O_2_^−^•production in HTN may partly involve PKC. In isolated arteries, high pressure induces O_2_^−^•production via PKC-dependent activation of NADPH oxidase [3]. Also, O_2_^−^•production is increased in sympathetic neurons of DOCA-salt hypertensive rats via activation of NADPH oxidase [116]. Studies have also shown that the impaired vasodilation and increased vascular O_2_^−^•production in the 2 kidney-1 clip (2K-1C) rat model of renovascular HTN are likely related to PKC-mediated activation of membrane-associated NADPH-dependent oxidase [2,3,117].

## 16. PKC, MMPs and Vascular Remodeling in Hypertension

PKC may play a signaling role in the expression and activity of MMPs and consequently affects ECM composition and vascular remodeling. MMPs are a family of zinc-containing proteases that play a role in the degradation of ECM proteins [118,119,120], and may have additional effects on the endothelium and VSM [121,122]. MMPs activity is regulated at the transcription level as well as by activation of their pro-form, interaction with specific ECM components, and inhibition by endogenous tissue inhibitors of MMPs (TIMPs). Changes in hemodynamics, vessel injury, inflammatory cytokines and ROS could upregulate MMPs and promote vascular remodeling and HTN. Some studies have shown that the plasma levels and activity of MMP-2, MMP-9 and TIMP-1 are elevated in hypertensive patients [123]. Other studies have shown that the plasma levels of active MMP-2 and -9 are decreased in patients with essential HTN, and treatment with amlodipine normalized MMP-9 plasma levels [124]. These findings suggested a relationship between abnormal ECM metabolism and HTN, and that antihypertensive treatment may modulate collagen metabolism. In a study examining the serum levels of carboxy-terminal telopeptide of collagen type I (CITP) as a marker of extracellular collagen type I degradation, MMP-1 (collagenase), TIMP-1, and MMP-1–TIMP-1 complex, baseline free MMP-1 was decreased and baseline free TIMP-1 was increased in hypertensive compared with normotensive subjects. Hypertensive patients treated with the angiotensin-converting enzyme (ACE) inhibitor lisinopril for 1 year showed an increase in free MMP-1, a decrease in free TIMP-1, and an increase in serum CITP. These findings suggest that systemic extracellular degradation of collagen type I is depressed in patients with essential HTN and may facilitate organ fibrosis, and this can be normalized by treatment with lisinopril [7]. Also, gelatin zymographic analysis of in internal mammary artery from normotensive and hypertensive patients undergoing coronary artery bypass surgery, indicated a decrease in activity of MMP-2 and -9 in HTN. MMP-1 activity was also decreased by 4-fold without a change in protein levels. Immunoblot analysis revealed a decrease in the tissue levels of extracellular matrix metalloproteinase inducer (EMMPRIN), MMP activator protein (MT1-MMP) and MMP-9 in HTN. Also, measurement of plasma markers of collagen synthesis (procollagen type I amino-terminal propeptide [PINP]) and collagen degradation (carboxy-terminal telopeptide of collagen type I [ICTP]) has shown no changes in PINP levels but decreased degradation of collagen in HTN. These data demonstrate that MMP-1 and -9, MMP inducer and activator proteins are downregulated in HTN, and may result in increased collagen deposition in HTN [8].

Studies have shown that the total wall thickness and the medial area are increased in the aorta but not vena cava of DOCA-salt *versus* sham rats. In HTN, MMP-2 expression and activity were increased in the aorta but not vena cava, while MMP-9 was weakly expressed in both vessels. TIMP-2 expression was increased in the aorta of DOCA-salt rats compared to sham, but barely detectable in vena cava of DOCA-salt and sham or rats. These data suggest a link between MMPs and vascular remodeling in the aorta of DOCA-salt hypertensive rats. The increase in TIMP-2 expression in the aorta of DOCA-salt rats may be an adaptive mechanism to the high levels of MMP-2 [9]. Other studies have shown that in wild-type mice treated with ANG II and a 5% NaCl diet for 10 days, the onset of HTN is accompanied by increased MMP-9 activity in conductance vessels. In contrast, in MMP-9^(−/−)^ mice, the absence of MMP-9 activity is associated with vessel stiffness and increased pulse pressure, suggesting that in early stages of HTN, MMP-9 activation may preserve vessel compliance and alleviate BP increase [125].

Growth factors and cytokines such as nuclear factor κB and IL-1α stimulate VSMCs to secrete MMP-1, -3, -9, and these effects may be dependent on activation of ζ-PKC, and may contribute to inhibition of VSMC proliferation and vascular remodeling [10]. PKC also increases MMP-2 secretion in endothelial cells [126], and PKC-α plays a critical role in MMP-9 secretion in bovine capillary endothelial cells through ERK1/2 signaling [11]. PKC-β plays a signaling role in the expression and activity of MMP-1 and -3 in human coronary artery endothelial cells [127]. In cardiac microvascular endothelial cells, IL-1β activates α-PKC and βI-PKC and increases the expression and activity of MMP-2, and inhibition of α-PKC and βI-PKC abrogates the IL-1β stimulated increase in MMP-2 [12].

## 17. PKC in Salt-Sensitive Hypertension

Increased dietary sodium intake causes HTN in salt-sensitive individuals [128,129]. Studies have shown an increase in BP and the heart to body weight ratio in DOCA salt-sensitive hypertensive rats compared to control rats. Also, α-, γ- and ε-PKC are upregulated while δ-PKC is not altered in cardiac extracts of DOCA-salt rats compared to controls. On the other hand, δ-PKC is increased in cardiac fibroblasts from DOCA-salt rats compared to controls. These data suggest cell-specific increase in the expression of α, γ, δ or ε-PKC in the hearts of DOCA-salt hypertensive rats [130]. Also, the PKC inhibitor GF109203X (2-[1-(3-dimethylaminopropyl)-1H-indol-3-yl]-3-(1H-indol-3-yl)maleimide) decreases both basal tone and MAPK (ERK1/2) activity in DOCA-salt rats, suggesting that the increased basal vascular tone and MAPK activity in DOCA-salt hypertensive rats may involve PKC [131].

Changes in cardiac PKC have also been observed in Dahl salt-sensitive hypertensive rats. Marinobufagenin, an endogenous ligand of the α1 subunit of the cardiac Na/K-ATPase, is increased in sodium-loaded Dahl-salt-sensitive rats, and PKC-induced phosphorylation of the α1 Na/K-ATPase may increase its sensitivity to marinobufagenin, and further contribute to the increased BP in this rat model [117].

PKC may also affect the renin-angiotensin-aldosterone system and the renal control mechanism of BP. Infusion of ANG II in rats causes HTN, vascular endothelial dysfunction and increased vascular O_2_^−^• production. Some of the vascular effects of ANG II may be mediated by increased endothelial cell release of ET-1, which in turn activates PKC [132,133,134]. Interestingly, ANG II-induced ET-1 production and PKC activity are greater in blood vessels of SHR than normotensive control rats [135]. Other studies have shown that cytosolic PKC activity is higher in aortic VSM from SHR than those from WKY or SHR treated with the angiotensin-converting enzyme (ACE) inhibitor enalapril, and the changes in vascular PKC activity were paralleled by changes in BP. Membrane-bound PKC activity was detected in aortic VSM of SHR, but not in that of the WKY or enalapril-treated SHR. Also, α-PKC mRNA expression and protein amount were greater in aortic VSM from SHR than those from WKY or enalapril-treated SHR, suggesting that the beneficial effects of ACE inhibitors in HTN may in part involve changes in expression and activity of α-PKC in VSM [136]. Other studies have shown that PKC could affect the Na^+^/Ca^2+^ exchange mechanism in the renal arterioles leading to defective renal vasodilation and salt-sensitive HTN [137].

PKC may also affect the renal tubular cells and the kidney function. In renal tubular epithelial cells, δ- and ζ-PKC are localized in the plasma membrane whereas α- and ε-PKC are cytosolic. Dopamine, an intrarenal modulator of sodium metabolism and BP, causes translocation of α- and ε-PKC to the plasma membrane [138,139], supporting a role of PKC in the control of renal sodium and water reabsorption and BP [140].

## 18. PKC, Neuronal Dysfunction and Hypertension

PKC may play a role in the neural control mechanisms of BP. The expression and redistribution of PKC isozymes are increased in brain tissue of SHR [141]. Also, sympathetic nerves are known to control VSM contraction by releasing chemical transmitters such as norepinephrine, which in turn trigger the increase in [Ca^2+^]_i_ and PKC activity. Polymorphisms in human tyrosine hydroxylase gene have been associated with increased sympathetic activity, norepinephrine release and HTN [142], and the role of PKC in these hypertensive subjects remains to be investigated.

## 19. PKC, Metabolic Dysfunction and Hypertension

Metabolic disorders are often associated with hyperglycemia and glucose intolerance, insulin resistance, central and overall obesity, dyslipidemia (increased triglyceride and decreased high-density lipoprotein (HDL) cholesterol levels), and different vascular manifestations and complications including HTN. Evidence suggests a role of PKC in these metabolic disorders. For example, glucose-induced increase in endothelial cell permeability is associated with activation of α-PKC [143]. Also, glucose, via activation of PKC, may affect the Na^+^/H^+^ exchanger mRNA expression and activity in VSMCs [144]. Importantly, an antisense complementary to the mRNA initiation codon regions for α- and β-PKC causes downregulation of these PKC isoforms and inhibits insulin-induced glucose uptake in rat adipocytes [145]. Also, inhibitors of β-PKC ameliorate the vascular dysfunction in rat models of diabetes and attenuate the progression of experimental diabetic nephropathy and HTN [146].

## 20. PKC, Vascular Inflammation and Hypertension

Vascular inflammation may play a role in cardiovascular disease [4,147]. Plasma levels of tumor necrosis factor-α (TNF-α), interleukin-1β (IL-1β), and IL-6 are increased in patients with HTN and coronary artery disease [5,6,148,149,150,151]. Also, infusion of ANG II does not induce HTN in IL-6 knockout mice, supporting a role of IL-6 in HTN [152]. In isolated pulmonary artery, hypoxia causes upregulation of TNF-α and IL-1β, a process that is dependent on PKC activation and promotes pulmonary vasoconstriction [13]. Also, TNF-α activates PKC and mitogenic signaling in cultured VSMCs [14], and inhibition of PKC-δ blocks high glucose-induced secretion of TNF-α in cultured rat and human aortic VSMCs [15].

## 21. PKC and Pulmonary Hypertension

PKC exert specific effects on the pulmonary vessels and may play a role in pulmonary HTN. Both insulin-like growth factor I and PKC activation stimulate proliferation of pulmonary artery VSMCs. PKC is also one of the signaling pathways involved in hypoxia-induced pulmonary artery VSMC proliferation, and chronic hypoxia may increase PKC activity and promote growth in pulmonary artery adventitial fibroblasts [153]. Mice deficient in ε-PKC show decreased hypoxic pulmonary vasoconstriction [154]. Also, ET-1 is a potent pulmonary vasoconstrictor, and endothelin receptor antagonists have shown benefits in patients with pulmonary HTN [155,156]. ET-1 induced pulmonary vasoconstriction is partly mediated by PKC, and PKC inhibitors decrease ET-1 induced pulmonary artery contraction [157]. 

## 22. PKC and Hypertension-in-Pregnancy and Preeclampsia

Normal pregnancy is often associated with decreased BP, increased uterine blood flow and decreased vascular responses to vasoconstrictors [158,159]. Uterine artery from pregnant sheep and aorta of late pregnant rats show decreased vascular contraction and PKC activity [160,161]. Also, the expression, activation and translocation of the Ca^2+^-dependent α-PKC and the Ca^2+^-independent δ- and ζ-PKC are reduced in the aorta of late pregnant compared with nonpregnant rats [161,162].

In 5% to 7% of pregnancies, women develop a condition called preeclampsia characterized by proteinuria and severe increases in BP [159]. Studies in animal models of HTN in pregnancy have provided useful information regarding the potential causes of preeclampsia. BP is greater in late pregnant rats treated with the NO synthase inhibitor l-NAME, compared with normal pregnant or virgin rats nontreated or treated with l-NAME [163]. Also, phenylephrine-induced contraction is greater in aortas from l-NAME-treated pregnant rats compared with normal pregnant or virgin rats [163,164]. Additionally, expression and activity of vascular α- and δ-PKC are enhanced in l-NAME-treated compared with non-treated pregnant rats [161,162], suggesting a role of α- and δ-PKC in the increased vasoconstriction and vascular resistance during HTN in pregnancy [161,162].

PKC may also play a role in the changes in ANG II receptor-mediated signaling during preeclampsia. In cultured neonatal rat cardiomyocytes, immunoglobulin from preeclamptic women enhances angiotensin type 1 (AT_1_) receptor-mediated chronotropic response, while immunoglobulin from control subjects has no effect, and the chronotropic effects of imunoglobulin are prevented by the PKC inhibitor calphostin C. Also, confocal microscopy of VSMCs has shown colocalization of purified IgG from preeclamptic women and AT_1_ receptor antibody. These findings have suggested that preeclamptic women develop auto-antibodies that stimulate AT_1_ receptor, a process that may to be mediated by PKC [165].

Experimental studies have suggested that reduction in uteroplacental perfusion pressure and the ensuing placental ischemia or hypoxia during late pregnancy may increase the release of cytokines into the maternal circulation, which in turn cause generalized vascular changes and HTN [159,166,167,168,169,170]. Plasma levels of TNF-α are elevated in women with preeclampsia [168,169]. Sources other than the placenta may also contribute to the elevated serum levels of TNF-α in preeclamptic women [171]. Interestingly, infusion of TNF-α or IL-6 in pregnant rats to reach plasma levels similar to those observed in preeclampsia, are associated with inceassed BP and systemic vasoconstriction [172,173]. Also, treatment of aortic segments from pregnant rats with TNF-α or IL-6 enhances reactivity to vasoconstrictor stimuli [174,175]. Cytokines may increase the expression and activity of vascular PKC leading to increased myofilament force sensitivity to [Ca^2+^]_i_ and enhanced VSM contraction. Other vasoactive factors such as soluble fms-like tyrosine kinase-1 (sFlt-1) and soluble endoglin (sEng) may be released during reduction of uteroplacental perfusion pressure [176,177] and their effects on PKC need to be examined.

## 23. PKC Inhibitors as Modulators of Vascular Function in Hypertension

The effects of PKC inhibitors on VSM contraction has been examined in isolated blood vessels, but the *in vivo* effects of PKC inhibitors have not been fully examined. Dahl-salt-sensitive rats on high NaCl (8%) diet exhibit an increase in BP, excretion of the endogenous inhibitor of α1 Na/K-ATPase marinobufagenin, left ventricular weight, and myocardial Na/K-ATPase and βII-PKC and δ-PKC. Treatment of Dahl-salt rats with cicletanine causes reduction in BP and left ventricular weight, decreased sensitivity of Na/K-ATPase to marinobufagenin, no increase in βII-PKC, and reduced phorbol diacetate-induced Na/K-ATPase phosphorylation. The cicletanine-induced decrease in BP may be due to targeting of PKC-induced phosphorylation of cardiac α1 Na/K-ATPase [117]. In isolated human mesenteric artery, marinobufagenin induces sustained vasoconstriction, possibly due to inhibition of the plasmalemmal Na/K-ATPase activity. Treatment of the vessel with cicletanine inhibited marinobufagenin-induced contraction and attenuated marinobufagenin-induced Na/K-ATPase inhibition, and the effects of cicletanine were prevented by the PKC activator phorbol diacetate. Similarly, in rat brain cicletanine inhibits PKC activity, and these inhibitory effects on PKC are prevented in the presence of phorbol diacetate. These data suggest that PKC is involved in the regulation of Na/K-ATPase and vascular tone, and may represent a potential target for therapeutic intervention in HTN [178].

It is important to note that HTN is a multifactorial disease, and PKC inhibitors alone may not be sufficient to manage HTN. However, PKC inhibitors may decrease the VSM growth and hyperactivity associated with HTN particularly when used with other therapeutic modalities. PKC inhibitors could potentiate the inhibitory effects of Ca^2+^ channel blockers on vasoconstriction. Targeting Ca^2+^-independent PKCs could be beneficial in Ca^2+^ antagonist-resistant forms of HTN. The effects of PKC inhibitors in reducing vasoconstriction and BP could also be potentiated by Rho-kinase and MAPK inhibitors. RhoA/Rho-kinase causes inhibition of MLC phosphatase and thereby enhances Ca^2+^-MLC kinase dependent VSM contraction, and may play a role in the development and progression of HTN [152,179]. The interaction between PKC and other pathways such as ROS, MMPs and inflammatory cytokines could also be associated with vascular disease. The combined use of isoform-specific PKC inhibitors with antioxidants, MMPs inhibitors and cytokine antagonists may provide a multi-prong approach for treatment of Ca^2+^ antagonist-insensitive forms of HTN.

Upregulation of PKC could play a role not only in vascular disease such as HTN and atherogenesis, but also in metabolic disorders, insulin resistance and cancer in what has been termed as the “PKC syndrome” [180]. Therefore, it is important to further test the effects of PKC inhibitors *in vivo* and in animal models of HTN with other co-morbidities such as hypercholesterolemia and diabetes. Although the first generation of PKC inhibitors may not be very selective, newly-developed PKC inhibitors are more specific, and further experimental studies and clinical trials are needed before these compounds can be used safely in human. Certain PKC inhibitors such as ruboxistaurin (LY333531), a selective β-PKC inhibitor, have shown promise in clinical trials for diabetic retinopathy, macular edema and microvascular complications [181,182,183]. Using similar strategies to develop specific inhibitors of α-, δ- or ε-PKC isoform with improved enzyme selectivity and pharmakokinetics may lead to new therapies for HTN.

## List of abbreviations

ANG II: angiotensin II
ATP: adenosine triphosphate
BP: blood pressure
CPI-17: PKC-potentiated phosphatase inhibitor protein-17 kDa
CAM: calmodulin
DAG: diacylglycerol
EC: endothelial cell
ET-1: endothelin-1
HTN: hypertension
IP_3_: inositol 1,4,5-trisphosphate
MAPK: mitogen-activated protein kinase
MARCKs: myristoylated alanine-rich C-kinase substrate
MMP: matrix metalloproteinase
MEK: MAPK kinase
MLC: myosin light chain
NADPH: nicotinamide adenine dinucleotide phosphate
O_2_^−^•: superoxide
PDBu: phorbol 12,13-dibutyrate
PIP_2_: phosphatidylinositol 4,5-bisphosphate
PLC: phospholipase C
PKC: protein kinase C
PMA: phorbol myristate acetate
RACKs: receptors for activated C-kinase
RAS: renin-angiotensin system
Rho-kinase: Rho-associated kinase
ROS: reactive oxygen species
SHR: spontaneously hypertensive rat
TPA: 12-o-tetradecanoylphorbol-13-acetate
VSMC: vascular smooth muscle cell
WKY: Wistar-Kyoto

## References

[1] Cardillo C., Kilcoyne C.M., Quyyumi A.A., Cannon R.O., Panza J.A. (1998). Selective defect in nitric oxide synthesis may explain the impaired endothelium-dependent vasodilation in patients with essential hypertension. Circulation.

[2] Heitzer T., Wenzel U., Hink U., Krollner D., Skatchkov M., Stahl R.A., MacHarzina R., Brasen J.H., Meinertz T., Munzel T. (1999). Increased NAD(P)H oxidase-mediated superoxide production in renovascular hypertension: evidence for an involvement of protein kinase C. Kidney Int..

[3] Ungvari Z., Csiszar A., Huang A., Kaminski P.M., Wolin M.S., Koller A. (2003). High pressure induces superoxide production in isolated arteries via protein kinase C-dependent activation of NAD(P)H oxidase. Circulation.

[4] Libby P. (2006). Inflammation and cardiovascular disease mechanisms. Am. J. Clin. Nutr..

[5] Nijm J., Wikby A., Tompa A., Olsson A.G., Jonasson L. (2005). Circulating levels of proinflammatory cytokines and neutrophil-platelet aggregates in patients with coronary artery disease. Am. J. Cardiol..

[6] McLachlan C.S., Chua W.C., Wong P.T., Kah T.L., Chen C., El Oakley R.M. (2005). Homocysteine is positively associated with cytokine IL-18 plasma levels in coronary artery bypass surgery patients. Biofactors.

[7] Laviades C., Varo N., Fernandez J., Mayor G., Gil M.J., Monreal I., Diez J. (1998). Abnormalities of the extracellular degradation of collagen type I in essential hypertension. Circulation.

[8] Ergul A., Portik-Dobos V., Hutchinson J., Franco J., Anstadt M.P. (2004). Downregulation of vascular matrix metalloproteinase inducer and activator proteins in hypertensive patients. Am. J. Hypertens..

[9] Watts S.W., Rondelli C., Thakali K., Li X., Uhal B., Pervaiz M.H., Watson R.E., Fink G.D. (2007). Morphological and biochemical characterization of remodeling in aorta and vena cava of DOCA-salt hypertensive rats. Am. J. Physiol. Heart Circ. Physiol..

[10] Hussain S., Assender J.W., Bond M., Wong L.F., Murphy D., Newby A.C. (2002). Activation of protein kinase Czeta is essential for cytokine-induced metalloproteinase-1, -3, and -9 secretion from rabbit smooth muscle cells and inhibits proliferation. J. Biol. Chem..

[11] Park M.J., Park I.C., Lee H.C., Woo S.H., Lee J.Y., Hong Y.J., Rhee C.H., Lee Y.S., Lee S.H., Shim B.S. (2003). Protein kinase C-alpha activation by phorbol ester induces secretion of gelatinase B/MMP-9 through ERK 1/2 pathway in capillary endothelial cells. Int. J. Oncol..

[12] Mountain D.J., Singh M., Menon B., Singh K. (2007). Interleukin-1beta increases expression and activity of matrix metalloproteinase-2 in cardiac microvascular endothelial cells: role of PKCalpha/beta1 and MAPKs. Am. J. Physiol. Cell. Physiol..

[13] Tsai B.M., Wang M., Pitcher J.M., Meldrum K.K., Meldrum D.R. (2004). Hypoxic pulmonary vasoconstriction and pulmonary artery tissue cytokine expression are mediated by protein kinase C. Am. J. Physiol. Lung Cell. Mol. Physiol..

[14] Ramana K.V., Chandra D., Srivastava S., Bhatnagar A., Srivastava S.K. (2003). Aldose reductase mediates the mitogenic signals of cytokines. Chem. Biol. Interact..

[15] Ramana K.V., Tammali R., Reddy A.B., Bhatnagar A., Srivastava S.K. (2007). Aldose reductase-regulated tumor necrosis factor-alpha production is essential for high glucose-induced vascular smooth muscle cell growth. Endocrinology.

[16] Somlyo A.P., Somlyo A.V. (2003). Ca^2+^ sensitivity of smooth muscle and nonmuscle myosin II: Modulated by G proteins, kinases, and myosin phosphatas. Physiol. Rev..

[17] Khalil R.A., van Breemen C. (1988). Sustained contraction of vascular smooth muscle: calcium influx or C-kinase activation?. J. Pharmacol. Exp. Ther..

[18] Horowitz A., Menice C.B., Laporte R., Morgan K.G. (1996). Mechanisms of smooth muscle contraction. Physiol. Rev..

[19] Salamanca D.A., Khalil R.A. (2005). Protein kinase C isoforms as specific targets for modulation of vascular smooth muscle function in hypertension. Biochem. Pharmacol..

[20] Berridge M.J., Irvine R.F. (1984). Inositol trisphosphate, a novel second messenger in cellular signal transduction. Nature.

[21] Nishizuka Y. (1992). Intracellular signaling by hydrolysis of phospholipids and activation of protein kinase C. Science.

[22] Morgan K.G., Khalil R.A., Suematsu E., Katsuyama H. (1992). Calcium-dependent and calcium-independent pathways of signal transduction in smooth muscle. Jpn. J. Pharmacol..

[23] Nishimura J., Khalil R.A., van Breemen C. (1989). Agonist-induced vascular tone. Hypertension.

[24] Jiang M.J., Morgan K.G. (1987). Intracellular calcium levels in phorbol ester-induced contractions of vascular muscle. Am. J. Physiol..

[25] Takai Y., Kishimoto A., Iwasa Y., Kawahara Y., Mori T., Nishizuka Y. (1979). Calcium-dependent activation of a multifunctional protein kinase by membrane phospholipids. J. Biol. Chem..

[26] Newton A.C. (1995). Protein kinase C: structure, function, and regulation. J. Biol. Chem..

[27] Klevit R.E., Herriott J.R., Horvath S.J. (1990). Solution structure of a zinc finger domain of yeast ADR1. Proteins.

[28] Coussens L., Parker P.J., Rhee L., Yang-Feng T.L., Chen E., Waterfield M.D., Francke U., Ullrich A. (1986). Multiple, distinct forms of bovine and human protein kinase C suggest diversity in cellular signaling pathways. Science.

[29] Parker C.A., Takahashi K., Tao T., Morgan K.G. (1994). Agonist-induced redistribution of calponin in contractile vascular smooth muscle cells. Am. J. Physiol..

[30] Ono Y., Fujii T., Ogita K., Kikkawa U., Igarashi K., Nishizuka Y. (1989). Protein kinase C zeta subspecies from rat brain: its structure, expression, and properties. Proc. Natl. Acad. Sci. USA.

[31] Ohno S., Konno Y., Akita Y., Yano A., Suzuki K. (1990). A point mutation at the putative ATP-binding site of protein kinase C alpha abolishes the kinase activity and renders it down-regulation-insensitive. A molecular link between autophosphorylation and down-regulation. J. Biol. Chem..

[32] Schaap D., Parker P.J., Bristol A., Kriz R., Knopf J. (1989). Unique substrate specificity and regulatory properties of PKC-epsilon: a rationale for diversity. FEBS Lett..

[33] Osada S., Mizuno K., Saido T.C., Suzuki K., Kuroki T., Ohno S. (1992). A new member of the protein kinase C family, nPKC theta, predominantly expressed in skeletal muscle. Mol. Cell. Biol..

[34] Bacher N., Zisman Y., Berent E., Livneh E. (1991). Isolation and characterization of PKC-L, a new member of the protein kinase C-related gene family specifically expressed in lung, skin, and heart. Mol. Cell. Biol..

[35] Haller H., Quass P., Lindschau C., Luft F.C., Distler A. (1994). Platelet-derived growth factor and angiotensin II induce different spatial distribution of protein kinase C-alpha and -beta in vascular smooth muscle cells. Hypertension.

[36] Singer H.A. (1990). Phorbol ester-induced stress and myosin light chain phosphorylation in swine carotid medial smooth muscle. J. Pharmacol. Exp. Ther..

[37] Ohanian V., Ohanian J., Shaw L., Scarth S., Parker P.J., Heagerty A.M. (1996). Identification of protein kinase C isoforms in rat mesenteric small arteries and their possible role in agonist-induced contraction. Circ. Res..

[38] Kanashiro C.A., Altirkawi K.A., Khalil R.A. (2000). Preconditioning of coronary artery against vasoconstriction by endothelin-1 and prostaglandin F2alpha during repeated downregulation of epsilon-protein kinase C. J. Cardiovasc. Pharmacol..

[39] Watanabe M., Hachiya T., Hagiwara M., Hidaka H. (1989). Identification of type III protein kinase C in bovine aortic tissue. Arch. Biochem. Biophys..

[40] Khalil R.A., Lajoie C., Morgan K.G. (1994). In situ determination of [Ca^2+^]i threshold for translocation of the alpha-protein kinase C isoform. Am. J. Physiol..

[41] Liou Y.M., Morgan K.G. (1994). Redistribution of protein kinase C isoforms in association with vascular hypertrophy of rat aorta. Am. J. Physiol..

[42] Khalil R.A., Lajoie C., Resnick M.S., Morgan K.G. (1992). Ca(2+)-independent isoforms of protein kinase C differentially translocate in smooth muscle. Am. J. Physiol..

[43] Goodnight J.A., Mischak H., Kolch W., Mushinski J.F. (1995). Immunocytochemical localization of eight protein kinase C isozymes overexpressed in NIH 3T3 fibroblasts. Isoform-specific association with microfilaments, Golgi, endoplasmic reticulum, and nuclear and cell membranes. J. Biol. Chem..

[44] Gailly P., Gong M.C., Somlyo A.V., Somlyo A.P. (1997). Possible role of atypical protein kinase C activated by arachidonic acid in Ca2+ sensitization of rabbit smooth muscle. J. Physiol..

[45] Makowske M., Rosen O.M. (1989). Complete activation of protein kinase C by an antipeptide antibody directed against the pseudosubstrate prototope. J. Biol. Chem..

[46] Orr J.W., Keranen L.M., Newton A.C. (1992). Reversible exposure of the pseudosubstrate domain of protein kinase C by phosphatidylserine and diacylglycerol. J. Biol. Chem..

[47] House C., Kemp B.E. (1987). Protein kinase C contains a pseudosubstrate prototope in its regulatory domain. Science.

[48] Dekker L.V., McIntyre P., Parker P.J. (1993). Mutagenesis of the regulatory domain of rat protein kinase C-eta. A molecular basis for restricted histone kinase activity. J. Biol. Chem..

[49] Kanashiro C.A., Khalil R.A. (1998). Signal transduction by protein kinase C in mammalian cells. Clin. Exp. Pharmacol. Physiol..

[50] Wang J.K., Walaas S.I., Sihra T.S., Aderem A., Greengard P. (1989). Phosphorylation and associated translocation of the 87-kDa protein, a major protein kinase C substrate, in isolated nerve terminals. Proc. Natl. Acad. Sci. USA.

[51] Hartwig J.H., Thelen M., Rosen A., Janmey P.A., Nairn A.C., Aderem A. (1992). MARCKS is an actin filament crosslinking protein regulated by protein kinase C and calcium-calmodulin. Nature.

[52] Katada T., Gilman A.G., Watanabe Y., Bauer S., Jakobs K.H. (1985). Protein kinase C phosphorylates the inhibitory guanine-nucleotide-binding regulatory component and apparently suppresses its function in hormonal inhibition of adenylate cyclase. Eur. J. Biochem..

[53] Barman S.A., Zhu S., White R.E. (2004). Protein kinase C inhibits BKCa channel activity in pulmonary arterial smooth muscle. Am. J. Physiol. Lung Cell. Mol. Physiol..

[54] Cogolludo A., Moreno L., Bosca L., Tamargo J., Perez-Vizcaino F. (2003). Thromboxane A2-induced inhibition of voltage-gated K+ channels and pulmonary vasoconstriction: role of protein kinase Czeta. Circ. Res..

[55] Limas C.J. (1980). Phosphorylation of cardiac sarcoplasmic reticulum by a calcium-activated, phospholipid-dependent protein kinase. Biochem. Biophys. Res. Commun..

[56] Rosoff P.M., Stein L.F., Cantley L.C. (1984). Phorbol esters induce differentiation in a pre-B-lymphocyte cell line by enhancing Na^+^/H^+^ exchange. J. Biol. Chem..

[57] Aviv A. (1994). Cytosolic Ca^2+^, Na^+^/H^+^ antiport, protein kinase C trio in essential hypertension. Am. J. Hypertens..

[58] Schwienbacher C., Jockusch B.M., Rudiger M. (1996). Intramolecular interactions regulate serine/threonine phosphorylation of vinculin. FEBS Lett..

[59] Woodsome T.P., Eto M., Everett A., Brautigan D.L., Kitazawa T. (2001). Expression of CPI-17 and myosin phosphatase correlates with Ca(2+) sensitivity of protein kinase C-induced contraction in rabbit smooth muscle. J. Physiol..

[60] Inagaki M., Yokokura H., Itoh T., Kanmura Y., Kuriyama H., Hidaka H. (1987). Purified rabbit brain protein kinase C relaxes skinned vascular smooth muscle and phosphorylates myosin light chain. Arch. Biochem. Biophys..

[61] Newton A.C. (1997). Regulation of protein kinase C. Curr. Opin. Cell. Biol..

[62] Mochly-Rosen D., Gordon A.S. (1998). Anchoring proteins for protein kinase C: A means for isozyme selectivity. FASEB J..

[63] Kraft A.S., Anderson W.B. (1983). Phorbol esters increase the amount of Ca^2+^, phospholipid-dependent protein kinase associated with plasma membrane. Nature.

[64] Hyatt S.L., Klauck T., Jaken S. (1990). Protein kinase C is localized in focal contacts of normal but not transformed fibroblasts. Mol. Carcinog..

[65] Kose A., Saito N., Ito H., Kikkawa U., Nishizuka Y., Tanaka C. (1988). Electron microscopic localization of type I protein kinase C in rat Purkinje cells. J. Neurosci..

[66] Cogolludo A., Moreno L., Lodi F., Tamargo J., Perez-Vizcaino F. (2005). Postnatal maturational shift from PKCzeta and voltage-gated K^+^ channels to RhoA/Rho kinase in pulmonary vasoconstriction. Cardiovasc. Res..

[67] Draeger A., Wray S., Babiychuk E.B. (2005). Domain architecture of the smooth-muscle plasma membrane: regulation by annexins. Biochem. J..

[68] Thelen M., Rosen A., Nairn A.C., Aderem A. (1991). Regulation by phosphorylation of reversible association of a myristoylated protein kinase C substrate with the plasma membrane. Nature.

[69] Cazaubon S.M., Parker P.J. (1993). Identification of the phosphorylated region responsible for the permissive activation of protein kinase C. J. Biol. Chem..

[70] Leventhal P.S., Bertics P.J. (1993). Activation of protein kinase C by selective binding of arginine-rich polypeptides. J. Biol. Chem..

[71] Ron D., Mochly-Rosen D. (1994). Agonists and antagonists of protein kinase C function, derived from its binding proteins. J. Biol. Chem..

[72] Housey G.M., Johnson M.D., Hsiao W.L., O'Brian C.A., Murphy J.P., Kirschmeier P., Weinstein I.B. (1988). Overproduction of protein kinase C causes disordered growth control in rat fibroblasts. Cell.

[73] Dallas A., Khalil R.A. (2003). Ca^2+^ antagonist-insensitive coronary smooth muscle contraction involves activation of epsilon-protein kinase C-dependent pathway. Am. J. Physiol. Cell. Physiol..

[74] Khalil R.A., Menice C.B., Wang C.L., Morgan K.G. (1995). Phosphotyrosine-dependent targeting of mitogen-activated protein kinase in differentiated contractile vascular cells. Circ. Res..

[75] Mii S., Khalil R.A., Morgan K.G., Ware J.A., Kent K.C. (1996). Mitogen-activated protein kinase and proliferation of human vascular smooth muscle cells. Am. J. Physiol..

[76] Adam L.P., Gapinski C.J., Hathaway D.R. (1992). Phosphorylation sequences in h-caldesmon from phorbol ester-stimulated canine aortas. FEBS Lett..

[77] D’Angelo G., Graceffa P., Wang C.A., Wrangle J., Adam L.P. (1999). Mammal-specific, ERK-dependent, caldesmon phosphorylation in smooth muscle. Quantitation using novel anti-phosphopeptide antibodies. J. Biol. Chem..

[78] Hedges J.C., Oxhorn B.C., Carty M., Adam L.P., Yamboliev I.A., Gerthoffer W.T. (2000). Phosphorylation of caldesmon by ERK MAP kinases in smooth muscle. Am. J. Physiol. Cell. Physiol..

[79] Bazzi M.D., Nelsestuen G.L. (1990). Protein kinase C interaction with calcium: A phospholipid-dependent process. Biochemistry.

[80] Nishizuka Y. (1995). Protein kinase C and lipid signaling for sustained cellular responses. FASEB J..

[81] Szallasi Z., Smith C.B., Pettit G.R., Blumberg P.M. (1994). Differential regulation of protein kinase C isozymes by bryostatin 1 and phorbol 12-myristate 13-acetate in NIH 3T3 fibroblasts. J. Biol. Chem..

[82] Giardina J.B., Tanner D.J., Khalil R.A. (2001). Oxidized-LDL enhances coronary vasoconstriction by increasing the activity of protein kinase C isoforms alpha and epsilon. Hypertension.

[83] Claro S., Kanashiro C.A., Oshiro M.E., Ferreira A.T., Khalil R.A. (2007). alpha- and epsilon-protein kinase C activity during smooth muscle cell apoptosis in response to gamma-radiation. J. Pharmacol. Exp. Ther..

[84] Li W., Zhang J., Bottaro D.P., Pierce J.H. (1997). Identification of serine 643 of protein kinase C-delta as an important autophosphorylation site for its enzymatic activity. J. Biol. Chem..

[85] Keranen L.M., Dutil E.M., Newton A.C. (1995). Protein kinase C is regulated in vivo by three functionally distinct phosphorylations. Curr. Biol..

[86] Edwards A.S., Newton A.C. (1997). Phosphorylation at conserved carboxyl-terminal hydrophobic motif regulates the catalytic and regulatory domains of protein kinase C. J. Biol. Chem..

[87] Eichholtz T., de Bont D.B., de Widt J., Liskamp R.M., Ploegh H.L. (1993). A myristoylated pseudosubstrate peptide, a novel protein kinase C inhibitor. J. Biol. Chem..

[88] Clement S., Tasinato A., Boscoboinik D., Azzi A. (1997). The effect of alpha-tocopherol on the synthesis, phosphorylation and activity of protein kinase C in smooth muscle cells after phorbol 12-myristate 13-acetate down-regulation. Eur. J. Biochem..

[89] Cain A.E., Khalil R.A. (2002). Pathophysiology of essential hypertension: role of the pump, the vessel, and the kidney. Semin. Nephrol..

[90] Wang S., Desai D., Wright G., Niles R.M., Wright G.L. (1997). Effects of protein kinase C alpha overexpression on A7r5 smooth muscle cell proliferation and differentiation. Exp. Cell. Res..

[91] Deng Z., Morse J.H., Slager S.L., Cuervo N., Moore K.J., Venetos G., Kalachikov S., Cayanis E., Fischer S.G., Barst R.J., Hodge S.E., Knowles J.A. (2000). Familial primary pulmonary hypertension (gene PPH1) is caused by mutations in the bone morphogenetic protein receptor-II gene. Am. J. Hum. Genet..

[92] Machado R.D., Pauciulo M.W., Thomson J.R., Lane K.B., Morgan N.V., Wheeler L., Phillips J.A., Newman J., Williams D., Galie N. (2001). BMPR2 haploinsufficiency as the inherited molecular mechanism for primary pulmonary hypertension. Am. J. Hum. Genet..

[93] Aldred M.A., Vijayakrishnan J., James V., Soubrier F., Gomez-Sanchez M.A., Martensson G., Galie N., Manes A., Corris P., Simonneau G. (2006). MPR2 gene rearrangements account for a significant proportion of mutations in familial and idiopathic pulmonary arterial hypertension. Hum. Mutat..

[94] Song Y., Jones J.E., Beppu H., Keaney J.F., Loscalzo J., Zhang Y.Y. (2005). Increased susceptibility to pulmonary hypertension in heterozygous BMPR2-mutant mice. Circulation.

[95] Hassel S., Eichner A., Yakymovych M., Hellman U., Knaus P., Souchelnytskyi S. (2004). Proteins associated with type II bone morphogenetic protein receptor (BMPR-II) and identified by two-dimensional gel electrophoresis and mass spectrometry. Proteomics.

[96] Shibata R., Morita S., Nagai K., Miyata S., Iwasaki T. (1990). Effects of H-7 (protein kinase inhibitor) and phorbol ester on aortic strips from spontaneously hypertensive rats. Eur. J. Pharmacol..

[97] Bazan E., Campbell A.K., Rapoport R.M. (1992). Protein kinase C activity in blood vessels from normotensive and spontaneously hypertensive rats. Eur. J. Pharmacol..

[98] Sauro M.D., Hadden J.W. (1992). Gamma-interferon corrects aberrant protein kinase C levels and immunosuppression in the spontaneously hypertensive rat. Int. J. Immunopharmacol..

[99] Sasajima H., Shima H., Toyoda Y., Kimura K., Yoshikawa A., Hano T., Nishio I. (1997). Increased Ca^2+^ sensitivity of contractile elements via protein kinase C in alpha-toxin permeabilized SMA from young spontaneously hypertensive rats. Cardiovasc. Res..

[100] Rosen B., Barg J., Zimlichman R. (1999). The effects of angiotensin II, endothelin-1, and protein kinase C inhibitor on DNA synthesis and intracellular calcium mobilization in vascular smooth muscle cells from young normotensive and spontaneously hypertensive rats. Am. J. Hypertens..

[101] Bilder G.E., Kasiewski C.J., Perrone M.H. (1990). Phorbol-12,13-dibutyrate-induced vasoconstriction in vivo: characterization of response in genetic hypertension. J. Pharmacol. Exp. Ther..

[102] Kanashiro C.A., Khalil R.A. (2001). Gender-related distinctions in protein kinase C activity in rat vascular smooth muscle. Am. J. Physiol. Cell. Physiol..

[103] Touyz R.M., Schiffrin E.L. (2001). Increased generation of superoxide by angiotensin II in smooth muscle cells from resistance arteries of hypertensive patients: role of phospholipase D-dependent NAD(P)H oxidase-sensitive pathways. J. Hypertens..

[104] Escriba P.V., Sanchez-Dominguez J.M., Alemany R., Perona J.S., Ruiz-Gutierrez V. (2003). Alteration of lipids, G proteins, and PKC in cell membranes of elderly hypertensives. Hypertension.

[105] Gu X., Bishop S.P. (1994). Increased protein kinase C and isozyme redistribution in pressure-overload cardiac hypertrophy in the rat. Circ. Res..

[106] Fatehi-Hassanabad Z., Fatehi M., Shahidi M.I. (2004). Endothelial dysfunction in aortic rings and mesenteric beds isolated from deoxycorticosterone acetate hypertensive rats: Possible involvement of protein kinase C. Eur. J. Pharmacol..

[107] Soloviev A.I., Parshikov A.V., Stefanov A.V. (1998). Evidence for the involvement of protein kinase C in depression of endothelium-dependent vascular responses in spontaneously hypertensive rats. J. Vasc. Res..

[108] Huang P.L., Huang Z., Mashimo H., Bloch K.D., Moskowitz M.A., Bevan J.A., Fishman M.C. (1995). Hypertension in mice lacking the gene for endothelial nitric oxide synthase. Nature.

[109] Michell B.J., Chen Z., Tiganis T., Stapleton D., Katsis F., Power D.A., Sim A.T., Kemp B.E. (2001). Coordinated control of endothelial nitric-oxide synthase phosphorylation by protein kinase C and the cAMP-dependent protein kinase. J. Biol. Chem..

[110] Fleming I., Fisslthaler B., Dimmeler S., Kemp B.E., Busse R. (2001). Phosphorylation of Thr(495) regulates Ca(2+)/calmodulin-dependent endothelial nitric oxide synthase activity. Circ. Res..

[111] Motley E.D., Eguchi K., Patterson M.M., Palmer P.D., Suzuki H., Eguchi S. (2007). Mechanism of endothelial nitric oxide synthase phosphorylation and activation by thrombin. Hypertension.

[112] Partovian C., Zhuang Z., Moodie K., Lin M., Ouchi N., Sessa W.C., Walsh K., Simons M. (2005). PKCalpha activates eNOS and increases arterial blood flow in vivo. Circ. Res..

[113] Vasquez-Vivar J., Kalyanaraman B., Martasek P., Hogg N., Masters B.S., Karoui H., Tordo P., Pritchard K.A. (1998). Superoxide generation by endothelial nitric oxide synthase: The influence of cofactors. Proc. Natl. Acad. Sci. USA.

[114] Xia Y., Tsai A.L., Berka V., Zweier J.L. (1998). Superoxide generation from endothelial nitric-oxide synthase. A Ca^2+^/calmodulin-dependent and tetrahydrobiopterin regulatory process. J. Biol. Chem..

[115] Li H., Witte K., August M., Brausch I., Godtel-Armbrust U., Habermeier A., Closs E.I., Oelze M., Munzel T., Forstermann U. (2006). Reversal of endothelial nitric oxide synthase uncoupling and up-regulation of endothelial nitric oxide synthase expression lowers blood pressure in hypertensive rats. J. Am. Coll. Cardiol..

[116] Dai X., Cao X., Kreulen D.L. (2006). Superoxide anion is elevated in sympathetic neurons in DOCA-salt hypertension via activation of NADPH oxidase. Am. J. Physiol. Heart Circ. Physiol..

[117] Fedorova O.V., Talan M.I., Agalakova N.I., Droy-Lefaix M.T., Lakatta E.G., Bagrov A.Y. (2003). Myocardial PKC beta2 and the sensitivity of Na/K-ATPase to marinobufagenin are reduced by cicletanine in Dahl hypertension. Hypertension.

[118] Galis Z.S., Khatri J.J. (2002). Matrix metalloproteinases in vascular remodeling and atherogenesis: the good, the bad, and the ugly. Circ. Res..

[119] Visse R., Nagase H. (2003). Matrix metalloproteinases and tissue inhibitors of metalloproteinases: structure, function, and biochemistry. Circ. Res..

[120] Benjamin M.M., Khalil R.A. (2012). Matrix metalloproteinase inhibitors as investigative tools in the pathogenesis and management of vascular disease. Experientia. Supplementum..

[121] Chew D.K., Conte M.S., Khalil R.A. (2004). Matrix metalloproteinase-specific inhibition of Ca^2+^ entry mechanisms of vascular contraction. J. Vasc. Surg..

[122] Raffetto J.D., Ross R.L., Khalil R.A. (2007). Matrix metalloproteinase 2-induced venous dilation via hyperpolarization and activation of K^+^ channels: Relevance to varicose vein formation. J. Vasc. Surg..

[123] Derosa G., D’Angelo A., Ciccarelli L., Piccinni M.N., Pricolo F., Salvadeo S., Montagna L., Gravina A., Ferrari I., Galli S. (2006). Matrix metalloproteinase-2, -9, and tissue inhibitor of metalloproteinase-1 in patients with hypertension. Endothelium.

[124] Zervoudaki A., Economou E., Stefanadis C., Pitsavos C., Tsioufis K., Aggeli C., Vasiliadou K., Toutouza M., Toutouzas P. (2003). Plasma levels of active extracellular matrix metalloproteinases 2 and 9 in patients with essential hypertension before and after antihypertensive treatment. J. Hum. Hypertens..

[125] Flamant M., Placier S., Dubroca C., Esposito B., Lopes I., Chatziantoniou C., Tedgui A., Dussaule J.C., Lehoux S. (2007). Role of matrix metalloproteinases in early hypertensive vascular remodeling. Hypertension.

[126] Papadimitriou E., Waters C.R., Manolopoulos V.G., Unsworth B.R., Maragoudakis M.E., Lelkes P.I. (2001). Regulation of extracellular matrix remodeling and MMP-2 activation in cultured rat adrenal medullary endothelial cells. Endothelium.

[127] Li D., Liu L., Chen H., Sawamura T., Ranganathan S., Mehta J.L. (2003). LOX-1 mediates oxidized low-density lipoprotein-induced expression of matrix metalloproteinases in human coronary artery endothelial cells. Circulation.

[128] Smith L., Payne J.A., Sedeek M.H., Granger J.P., Khalil R.A. (2003). Endothelin-induced increases in Ca^2+^ entry mechanisms of vascular contraction are enhanced during high-salt diet. Hypertension.

[129] Khalil R.A. (2006). Dietary salt and hypertension: new molecular targets add more spice. Am. J. Physiol. Regul. Integr. Comp. Physiol..

[130] Fareh J., Touyz R.M., Schiffrin E.L., Thibault G. (2000). Altered cardiac endothelin receptors and protein kinase C in deoxycorticosterone-salt hypertensive rats. J. Mol. Cell. Cardiol..

[131] Kim J., Lee Y.R., Lee C.H., Choi W.H., Lee C.K., Bae Y.M., Cho S., Kim B. (2005). Mitogen-activated protein kinase contributes to elevated basal tone in aortic smooth muscle from hypertensive rats. Eur. J. Pharmacol..

[132] Sirous Z.N., Fleming J.B., Khalil R.A. (2001). Endothelin-1 enhances eicosanoids-induced coronary smooth muscle contraction by activating specific protein kinase C isoforms. Hypertension.

[133] Cain A.E., Tanner D.M., Khalil R.A. (2002). Endothelin-1--induced enhancement of coronary smooth muscle contraction via MAPK-dependent and MAPK-independent [Ca(2+)](i) sensitization pathways. Hypertension.

[134] Khalil R.A. (2011). Modulators of the vascular endothelin receptor in blood pressure regulation and hypertension. Curr. Mol. Pharmacol..

[135] Schiffrin E.L. (1995). Endothelin: potential role in hypertension and vascular hypertrophy. Hypertension.

[136] Kanayama Y., Negoro N., Okamura M., Konishi Y., Nishimura M., Umetani N., Inoue T., Takeda T. (1994). Modulation of protein kinase C in aorta of spontaneously hypertensive rats with enalapril treatment. Osaka City Med. J..

[137] Bell P.D., Mashburn N., Unlap M.T. (2000). Renal sodium/calcium exchange; a vasodilator that is defective in salt-sensitive hypertension. Acta. Physiol. Scand..

[138] Nowicki S., Kruse M.S., Brismar H., Aperia A. (2000). Dopamine-induced translocation of protein kinase C isoforms visualized in renal epithelial cells. Am. J. Physiol. Cell. Physiol..

[139] Ridge K.M., Dada L., Lecuona E., Bertorello A.M., Katz A.I., Mochly-Rosen D., Sznajder J.I. (2002). Dopamine-induced exocytosis of Na,K-ATPase is dependent on activation of protein kinase C-epsilon and -delta. Mol. Biol. Cell..

[140] Banday A.A., Fazili F.R., Lokhandwala M.F. (2007). Oxidative stress causes renal dopamine D1 receptor dysfunction and hypertension via mechanisms that involve nuclear factor-kappaB and protein kinase C. J. Am. Soc. Nephrol..

[141] Hughes-Darden C.A., Wachira S.J., Denaro F.J., Taylor C.V., Brunson K.J., Ochillo R., Robinson T.J.  (2001). Expression and distribution of protein kinase C isozymes in brain tissue of spontaneous hypertensive rats. Cell. Mol. Biol. (Noisy-le-grand).

[142] Rao F., Zhang L., Wessel J., Zhang K., Wen G., Kennedy B.P., Rana B.K., Das M., Rodriguez-Flores J.L., Smith D.W. (2007). Tyrosine hydroxylase, the rate-limiting enzyme in catecholamine biosynthesis: discovery of common human genetic variants governing transcription, autonomic activity, and blood pressure in vivo. Circulation.

[143] Hempel A., Maasch C., Heintze U., Lindschau C., Dietz R., Luft F.C., Haller H. (1997). High glucose concentrations increase endothelial cell permeability via activation of protein kinase C alpha. Circ. Res..

[144] Williams B., Howard R.L. (1994). Glucose-induced changes in Na^+^/H^+^ antiport activity and gene expression in cultured vascular smooth muscle cells. Role of protein kinase C. J. Clin. Invest..

[145] Farese R.V., Standaert M.L., Ishizuka T., Yu B., Hernandez H., Waldron C., Watson J., Farese J.P., Cooper D.R., Wickstrom E. (1991). Antisense DNA downregulates protein kinase C isozymes (beta and alpha) and insulin-stimulated 2-deoxyglucose uptake in rat adipocytes. Antisense Res. Dev..

[146] Ishii H., Jirousek M.R., Koya D., Takagi C., Xia P., Clermont A., Bursell S.E., Kern T.S., Ballas L.M., Heath W.F. (1996). Amelioration of vascular dysfunctions in diabetic rats by an oral PKC beta inhibitor. Science.

[147] Young J.L., Libby P., Schonbeck U. (2002). Cytokines in the pathogenesis of atherosclerosis. Thromb. Haemost..

[148] Waehre T., Yndestad A., Smith C., Haug T., Tunheim S.H., Gullestad L., Froland S.S., Semb A.G., Aukrust P., Damas J.K. (2004). Increased expression of interleukin-1 in coronary artery disease with downregulatory effects of HMG-CoA reductase inhibitors. Circulation.

[149] Sardella G., Mariani P., D’Alessandro M., De Luca L., Pierro M., Mancone M., Porretta A., Accapezzato D., Fedele F., Paroli M. (2006). Early elevation of interleukin-1beta and interleukin-6 levels after bare or drug-eluting stent implantation in patients with stable angina. Thromb. Res..

[150] Lubrano V., Cocci F., Battaglia D., Papa A., Marraccini P., Zucchelli G.C. (2005). Usefulness of high-sensitivity IL-6 measurement for clinical characterization of patients with coronary artery disease. J. Clin. Lab. Anal..

[151] Funayama H., Ishikawa S.E., Kubo N., Katayama T., Yasu T., Saito M., Kawakami M. (2004). Increases in interleukin-6 and matrix metalloproteinase-9 in the infarct-related coronary artery of acute myocardial infarction. Circ. J..

[152] Lee D.L., Sturgis L.C., Labazi H., Osborne J.B., Fleming C., Pollock J.S., Manhiani M., Imig J.D., Brands M.W. (2006). Angiotensin II hypertension is attenuated in interleukin-6 knockout mice. Am. J. Physiol. Heart Circ. Physiol..

[153] Das M., Dempsey E.C., Bouchey D., Reyland M.E., Stenmark K.R. (2000). Chronic hypoxia induces exaggerated growth responses in pulmonary artery adventitial fibroblasts: potential contribution of specific protein kinase c isozymes. Am. J. Respir. Cell. Mol. Biol..

[154] Littler C.M., Morris K.G., Fagan K.A., McMurtry I.F., Messing R.O., Dempsey E.C. (2003). Protein kinase C-epsilon-null mice have decreased hypoxic pulmonary vasoconstriction. Am. J. Physiol. Heart Circ. Physiol..

[155] Ito T., Ozawa K., Shimada K. (2007). Current drug targets and future therapy of pulmonary arterial hypertension. Curr. Med. Chem..

[156] Puri A., McGoon M.D., Kushwaha S.S. (2007). Pulmonary arterial hypertension: current therapeutic strategies. Nat. Clin. Pract. Cardiovasc. Med..

[157] Barman S.A. (2007). Vasoconstrictor effect of endothelin-1 on hypertensive pulmonary arterial smooth muscle involves Rho-kinase and protein kinase C. Am. J. Physiol. Lung Cell. Mol. Physiol..

[158] Khalil R.A., Granger J.P. (2002). Vascular mechanisms of increased arterial pressure in preeclampsia: Lessons from animal models. Am. J. Physiol. Regul. Integr. Comp. Physiol..

[159] Sheppard S.J., Khalil R.A. (2010). Risk factors and mediators of the vascular dysfunction associated with hypertension in pregnancy. Cardiovasc. Hematol. Disord. Drug Targets.

[160] Magness R.R., Rosenfeld C.R., Carr B.R. (1991). Protein kinase C in uterine and systemic arteries during ovarian cycle and pregnancy. Am. J. Physiol..

[161] Kanashiro C.A., Cockrell K.L., Alexander B.T., Granger J.P., Khalil R.A. (2000). Pregnancy-associated reduction in vascular protein kinase C activity rebounds during inhibition of NO synthesis. Am. J. Physiol. Regul. Integr. Comp. Physiol..

[162] Kanashiro C.A., Alexander B.T., Granger J.P., Khalil R.A. (1999). Ca(2+)-insensitive vascular protein kinase C during pregnancy and NOS inhibition. Hypertension.

[163] Khalil R.A., Crews J.K., Novak J., Kassab S., Granger J.P. (1998). Enhanced vascular reactivity during inhibition of nitric oxide synthesis in pregnant rats. Hypertension.

[164] Crews J.K., Novak J., Granger J.P., Khalil R.A. (1999). Stimulated mechanisms of Ca^2+^ entry into vascular smooth muscle during NO synthesis inhibition in pregnant rats. Am. J. Physiol..

[165] Wallukat G., Homuth V., Fischer T., Lindschau C., Horstkamp B., Jupner A., Baur E., Nissen E., Vetter K., Neichel D. (1999). Patients with preeclampsia develop agonistic autoantibodies against the angiotensin AT1 receptor. J. Clin. Invest..

[166] Kupferminc M.J., Peaceman A.M., Wigton T.R., Rehnberg K.A., Socol M.L.  (1994). Tumor necrosis factor-alpha is elevated in plasma and amniotic fluid of patients with severe preeclampsia. Am. J. Obstet. Gynecol..

[167] Vince G.S., Starkey P.M., Austgulen R., Kwiatkowski D., Redman C.W. (1995). Interleukin-6, tumour necrosis factor and soluble tumour necrosis factor receptors in women with pre-eclampsia. Br. J. Obstet. Gynaecol..

[168] Conrad K.P., Benyo D.F. (1997). Placental cytokines and the pathogenesis of preeclampsia. Am. J. Reprod. Immunol..

[169] Williams M.A., Mahomed K., Farrand A., Woelk G.B., Mudzamiri S., Madzime S., King I.B., McDonald G.B. (1998). Plasma tumor necrosis factor-alpha soluble receptor p55 (sTNFp55) concentrations in eclamptic, preeclamptic and normotensive pregnant Zimbabwean women. J. Reprod. Immunol..

[170] LaMarca B.D., Ryan M.J., Gilbert J.S., Murphy S.R., Granger J.P. (2007). Inflammatory cytokines in the pathophysiology of hypertension during preeclampsia. Curr. Hypertens. Rep..

[171] Benyo D.F., Smarason A., Redman C.W., Sims C., Conrad K.P. (2001). Expression of inflammatory cytokines in placentas from women with preeclampsia. J. Clin. Endocrinol. Metab..

[172] Davis J.R., Giardina J.B., Green G.M., Alexander B.T., Granger J.P., Khalil R.A. (2002). Reduced endothelial NO-cGMP vascular relaxation pathway during TNF-alpha-induced hypertension in pregnant rats. Am. J. Physiol. Regul. Integr. Comp. Physiol..

[173] Orshal J.M., Khalil R.A. (2004). Reduced endothelial NO-cGMP-mediated vascular relaxation and hypertension in IL-6-infused pregnant rats. Hypertension.

[174] Giardina J.B., Green G.M., Cockrell K.L., Granger J.P., Khalil R.A. (2002). TNF-alpha enhances contraction and inhibits endothelial NO-cGMP relaxation in systemic vessels of pregnant rats. Am. J. Physiol. Regul. Integr. Comp. Physiol..

[175] Orshal J.M., Khalil R.A. (2004). Interleukin-6 impairs endothelium-dependent NO-cGMP-mediated relaxation and enhances contraction in systemic vessels of pregnant rats. Am. J. Physiol. Regul. Integr. Comp. Physiol..

[176] Gilbert J.S., Babcock S.A., Granger J.P. (2007). Hypertension produced by reduced uterine perfusion in pregnant rats is associated with increased soluble fms-like tyrosine kinase-1 expression. Hypertension.

[177] Gilbert J.S., Gilbert S.A., Arany M., Granger J.P. (2009). Hypertension produced by placental ischemia in pregnant rats is associated with increased soluble endoglin expression. Hypertension.

[178] Bagrov A.Y., Dmitrieva R.I., Dorofeeva N.A., Fedorova O.V., Lopatin D.A., Lakatta E.G., Droy-Lefaix M.T. (2000). Cicletanine reverses vasoconstriction induced by the endogenous sodium pump ligand, marinobufagenin, via a protein kinase C dependent mechanis. J. Hypertens..

[179] Seko T., Ito M., Kureishi Y., Okamoto R., Moriki N., Onishi K., Isaka N., Hartshorne D.J., Nakano T. (2003). Activation of RhoA and inhibition of myosin phosphatase as important components in hypertension in vascular smooth muscle. Circ. Res..

[180] McCarty M.F. (1996). Up-regulation of intracellular signalling pathways may play a central pathogenic role in hypertension, atherogenesis, insulin resistance, and cancer promotion—The “PKC syndrome”. Med. Hypotheses.

[181] Davis M.D., Sheetz M.J., Aiello L.P., Milton R.C., Danis R.P., Zhi X., Girach A., Jimenez M.C., Vignati L. (2009). Effect of ruboxistaurin on the visual acuity decline associated with long-standing diabetic macular edema. Invest. Ophthalmol. Vis. Sci..

[182] Aiello L.P., Vignati L., Sheetz M.J., Zhi X., Girach A., Davis M.D., Wolka A.M., Shahri N., Milton R.C. (2011). Oral protein kinase C beta inhibition using ruboxistaurin: efficacy, safety, and causes of vision loss among 813 patients (1,392 eyes) with diabetic retinopathy in the protein kinase C beta inhibitor-diabetic retinopathy study and the protein kinase C beta inhibitor-diabetic retinopathy study 2. Retina.

[183] Joy S.V., Scates A.C., Bearelly S., Dar M., Taulien C.A., Goebel J.A., Cooney M.J. (2005). Ruboxistaurin, a protein kinase C beta inhibitor, as an emerging treatment for diabetes microvascular complications. Ann. Pharmacother..

